# A parallel genome-wide mRNA and microRNA profiling of the frontal cortex of HIV patients with and without HIV-associated dementia shows the role of axon guidance and downstream pathways in HIV-mediated neurodegeneration

**DOI:** 10.1186/1471-2164-13-677

**Published:** 2012-11-28

**Authors:** Li Zhou, Gulietta M Pupo, Priyanka Gupta, Bing Liu, Sieu L Tran, Raany Rahme, Bin Wang, Rejane Rua, Helen Rizos, Adam Carroll, Murray J Cairns, Nitin K Saksena

**Affiliations:** 1 Retroviral Genetics Division, Center for Virus Research, Westmead Millennium Institute, Westmead Hospital, The University of Sydney, Westmead, NSW 2145, Sydney, Australia; 2 Westmead Institute for Cancer Research, University of Sydney at Westmead Millennium Institute, Westmead, NSW, 2145, Australia; 3 School of Biomedical Sciences and Pharmacy, Faculty of Health and the Hunter Medical Research Institute, The University of Newcastle, University Drive, Callaghan, NSW, 2308, Australia; 4 Schizophrenia Research Institute, Darlinghurst, Sydney NSW, Australia

**Keywords:** HIV-associated dementia, Neurodegeneration, HIV, Microarray, MicroRNA, Axon guidance

## Abstract

**Background:**

HIV-associated dementia (HAD) is the most common dementia type in young adults less than 40 years of age. Although the neurotoxins, oxidative/metabolic stress and impaired activity of neurotrophic factors are believed to be underlying reasons for the development of HAD, the genomic basis, which ultimately defines the virus-host interaction and leads to neurologic manifestation of HIV disease is lacking. Therefore, identifying HIV fingerprints on the host gene machinery and its regulation by microRNA holds a great promise and potential for improving our understanding of HAD pathogenesis, its diagnosis and therapy.

**Results:**

A parallel profiling of mRNA and miRNA of the frontal cortex autopsies from HIV positive patients with and without dementia was performed using Illumina Human-6 BeadChip and Affymetrix version 1.0 miRNA array, respectively. The gene ontology and pathway analysis of the two data sets showed high concordance between miRNA and mRNAs, revealing significant interference with the host axon guidance and its downstream signalling pathways in HAD brains. Moreover, the differentially expressed (DE) miRNAs identified in this study, in particular miR-137, 153 and 218, based on which most correlations were built cumulatively targeted neurodegeneration related pathways, implying their future potential in diagnosis, prognosis and possible therapies for HIV-mediated and possibly other neurodegenerative diseases. Furthermore, this relationship between DE miRNAs and DE mRNAs was also reflected in correlation analysis using Bayesian networks by splitting-averaging strategy (SA-BNs), which revealed 195 statistically significant correlated miRNA-mRNA pairs according to Pearson’s correlation test (P<0.05).

**Conclusions:**

Our study provides the first evidence on unambiguous support for intrinsic functional relationship between mRNA and miRNA in the context of HIV-mediated neurodegeneration, which shows that neurologic manifestation in HIV patients possibly occurs through the interference with the host axon guidance and its downstream signalling pathways. These data provide an excellent avenue for the development of new generation of diagnostic/prognostic biomarkers and therapeutic intervention strategies for HIV-associated neurodegeneration.

## Background

HIV-associated dementia (HAD) is the most common dementia type in young adults less than 40 years of age. To date, the cumulative incidence of HAD is 25-38%, and the prevalence is around 37% [1]. Although the highly active antiretroviral therapy (HAART) has had considerable success in preventing virus-mediated immune collapse end-stage complications, the prevalence of HIV-associated cognitive impairment appears to be on the rise due to the increased life span of HIV+ population.

It is well recognized that host-virus interactions play a crucial role in the occurrence and pathogenesis of HAD. Microarray and high throughput genomic technologies have greatly facilitated the examination of this interaction. A panoply of host genes have been shown to be influenced by HIV infection that facilitate subversion and manipulation of the host immune system during HIV infection of the brain. To date, most studies exploring HAD pathogenesis [2-6] have largely been confined to cultured cells, cerebrospinal fluid (CSF) and animal models, which can’t mimic and reveal the breadth of *in vivo* cellular responses subverted and manipulated as a consequence of HIV infection.

Recently, microRNAs (miRNAs), a class of small (20–25 nucleotides) non-coding RNAs, have been recognized as master post-transcriptional regulators of mRNAs [7] due to their numerous targeting capabilities along with their ability to form non-linear functionally viable gene regulatory networks, which together have wide-ranging effects on the control of host gene expression. In addition, miRNAs are also involved in diverse processes, which include neuronal development, cell differentiation, synapse formation and neuronal plasticity [8,9]. Thus, not surprisingly, miRNAs are significantly involved in neurodegenerative diseases, such as Parkinson disease (PD) [10], Alzheimer’s disease (AD) [11], Schizophrenia [12-15], aggressive behaviour [16] and depression [17]. Further, miRNAs also play an important role in HIV-host interaction [18]. HIV can even manipulate the expression of host neuronal genes, such as SNAP25 and cell death-related genes, including caspase-6, via the modulation of expression of selected microRNAs, which might serve as key elements in gene regulatory networks in HIV-associated neurobehavioral disorder [19-21].

To date, a joint study of the genome wide mRNA and miRNA profiling based on HIV-infected brain tissue with and without dementia is still lacking, although some individual mRNA [22-25] and miRNA [26] profiling studies based on human brain have been done. Therefore, our innovative approach has studied the utility of parallel genome-wide mRNA and miRNA analysis in the native frontal lobe post-mortem brain tissue from HIV patients with and without dementia. This carries enormous value in understanding gene expression in the context of HIV-mediated neurodegeneration and its regulation through miRNAs. This study has been designed with an objective to comprehensively delineate host transcriptional programming that occurs in concert with the regulatory miRNAs and to find how this interaction between mRNA and miRNA tampers with neurodegenerative pathways and dictates neurological manifestation of HIV disease. Here, we examined the gene ontology and pathways of differentially expressed (DE) mRNA in parallel with the global gene targets of DE miRNAs. Moreover, we derived paired functional correlation between mRNA and miRNA expressions using splitting-averaging strategy (SA-BNs) to demonstrate intrinsic functional relationship between gene expression and its regulation. In light of these data we believe that these results will not only facilitate a greater understanding of HIV pathogenesis of the brain and its neurological manifestation, but will also help define potential candidates for early detection and future therapy for neurodegeneration in HIV patients and related disorders.

## Results

### A snapshot of DE mRNA and miRNA profiles between HAD and HIV non-demented brains

In order to identify the mRNA and miRNA, which may contribute to the pathogenesis of HAD, a parallel genome-wide mRNA and miRNA profiling of the frontal cortex from HIV patients with and without dementia was performed.

GenomeStudio was used to analyse the normalized mRNA dataset. We observed 468 statistically significant candidate genes that were differentially expressed between two groups (P<0.05) (see Additional file 1). Among them, 432 genes were down regulated and 36 genes were up regulated (P<0.05), and 203 of 432 genes dysregulated greater than 1.5-fold change. To determine the similarity of global gene expression between samples and also in relation to the DE genes, hierarchical clustering was performed and generated using GenomeStudio (Additional file 2A). The HAD group formed an independent cluster away from the HIV non-dementia group, with the exception of one sample with dementia (neurological status less than 2) which clustered together with the HIV non-dementia group. Not surprisingly, the HIV non-dementia group clustered together with HIV negative control due to the absence of neuropathological changes and the absence of actively replicating HIV, which is consistent with a previous study [27] and our own study [28].

The analysis of miRNA data using GeneSpring identified 68 miRNA that were significantly differentially expressed in HAD and HIV non-dementia cortex (Additional file 3). Among them, 49 miRNAs were down regulated and 19 were upregulated. 27/68 miRNAs were dysregulated greater than 2 fold. Hierarchical clustering was carried out to demonstrate patterns of miRNA expression profiling between two groups (Additional file 2B), which was consistent with our mRNA hierarchical clustering. TargetScan was used to predict the gene targets of DE miRNAs, because it is the most advanced, respected, widely used and relatively conservative database in comparison to other databases (Additional file 4). In addition, we have carried out a low and high stringency G-seed search for miR-137, miR-153 and miR-218 targets based on a minimal free energy (MFE) ¾ -10 and ¾-14, respectively (Additional file 5). Furthermore, these predictions were made in the context of changes in gene expression observed in the same RNA.

**Figure 1:**
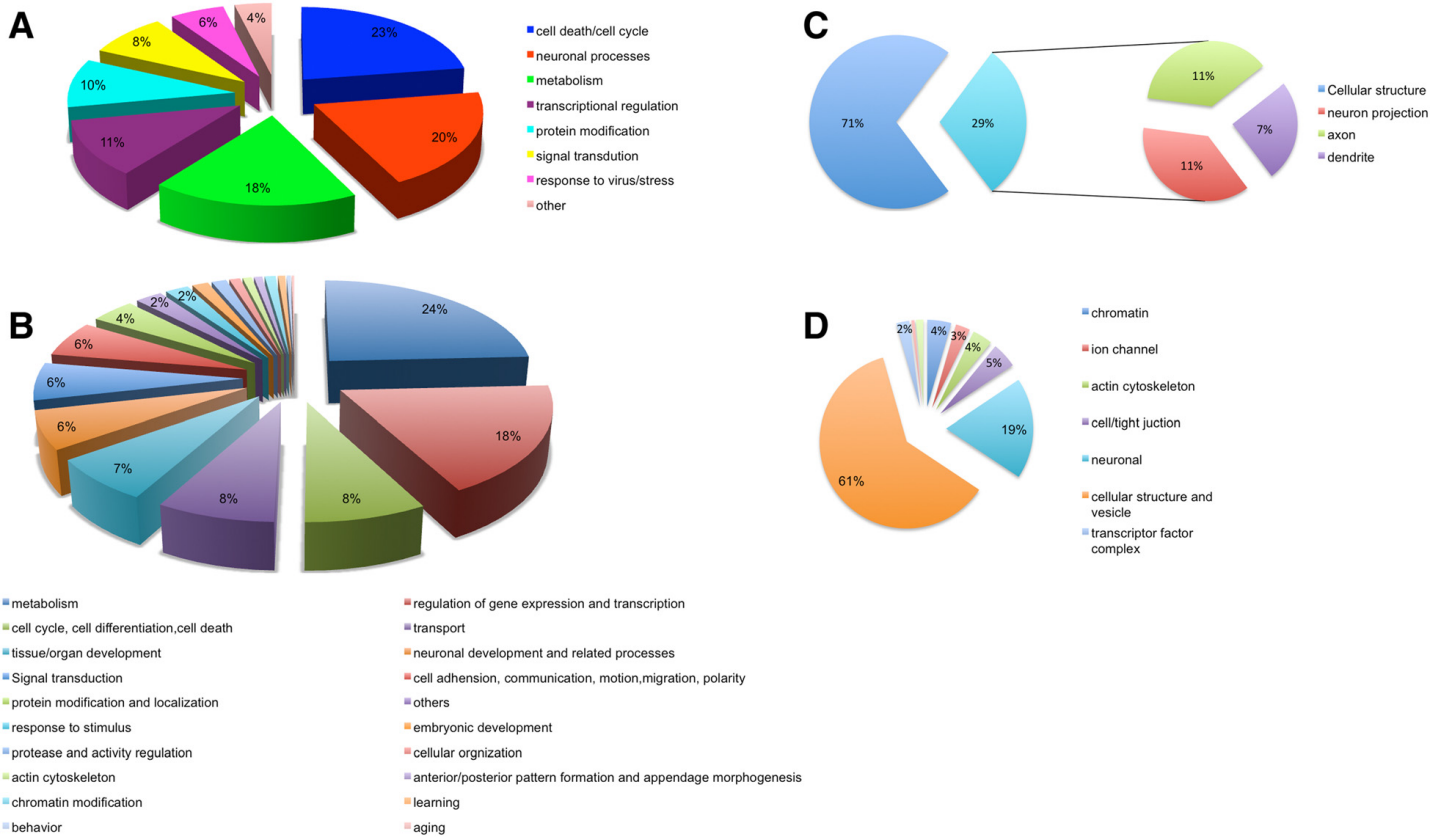
**Representation of gene ontology cellular components and biological processes of mRNA and miRNA profiles.** A pie chart representation of biological processes and cellular components, where each part represents -log_2_ of the P-value of biological process and cellular component from the set of significant biological processes and cellular components. The total of -log_2_ of the P-value is 1. The P-values were retrieved from gene ontology analysis in DAVID. **A**. Biological processes of mRNA profiles. **B**. Biological processes of miRNA profiles. **C**. Cellular components of mRNA profiles. **D**. Cellular components of miRNA profiles.

**Figure 2:**
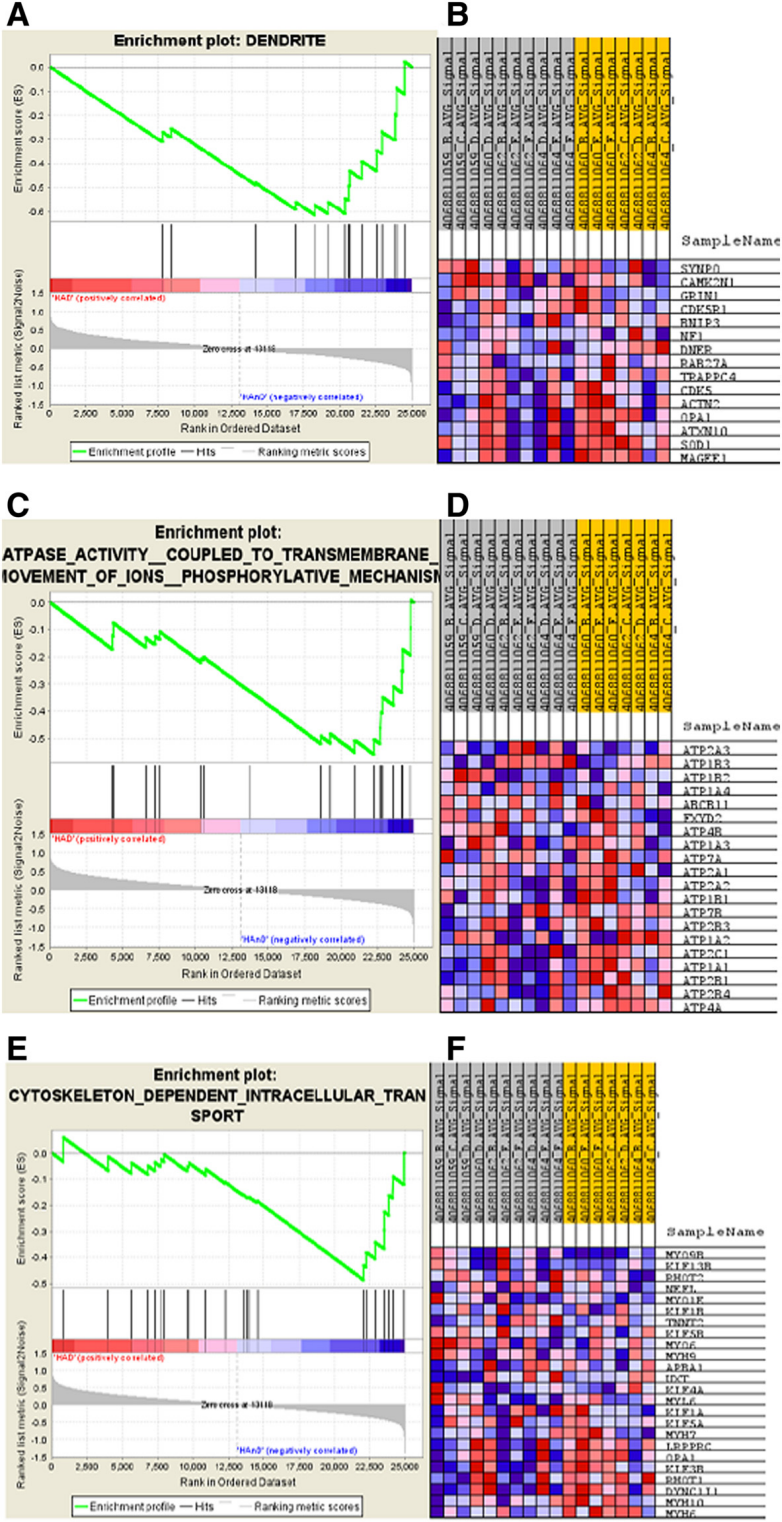
**Enrichment plots and heat maps for the gene sets of dendrite, ATPase activity coupled to ion transmembrane movement and cytoskeleton dependent intracellular transport generated by GSEA. **(**A**) Enrichment plot for the gene set of dendrite. Y-axis: value of the ranking metric; X-axis: the rank for all genes. Bottom: plot of the ranked list of all genes. Genes whose expression levels are most closely associated with the HAD or HIV non-dementia group are located at the left or right edge of the list while genes from the gene set dendrite within the ranked list are located at the middle. Top: the running enrichment score for the gene set as the analysis walks along the ranked list. The score at the peak of the plot is the enrichment score (ES) for this gene set and those genes appear before or at the peak are defined as core enrichment genes for this gene set. (**B**) Heat map of the core enrichment genes of the dendrite gene set, which contributes most to the ES. Rows: gene; columns: samples. Range of colors (red to blue) indicates the range of expression values (high to low). (**C**) Enrichment plot for the gene set of ATPase activity coupled to ion transmembrane movement. (**D**) Heat map of the core enrichment genes of the gene set ATPase activity coupled to ion transmembrane movement. (**E**) Enrichment plot for the gene set of cytoskeleton dependent intracellular transport. (**F**) Heat map of the core enrichment genes of the gene set cytoskeleton dependent intracellular transport.

Functional annotation of the mRNA DE genes and miRNA target genes was carried out in Database for Annotation, Visualization and Integrated Discovery (DAVID) together with extensive literature search. These mRNA DE genes were significantly associated with biological processes, such as cell death/cell cycle, neuronal processes, metabolism, transcriptional regulation, protein modification, signal transduction, and response to virus/stress, as shown in Figure 1A. In addition, according to cellular components distribution, 29% of genes fell into neuronal-related components, such as axon, neuron projection, and dendrite (Figure 1C). Furthermore, Gene Set Enrichment Analysis (GSEA) was also used to examine significantly enriched GO (gene ontology) gene sets comparing the normalized data of the entire 48,701 gene transcripts from HAD and HIV non-dementia brains to 1454 GO gene sets in GSEA Molecular Signatures Database (MsigDB) [29]. Eight gene sets were found statistical significantly enriched (P<0.05) in HIV non-dementia group while no gene sets were significantly enriched in the HAD group (indicating down regulation of those 8 gene sets in the HAD group) (Additional file 6). Of the eight enriched gene sets in the non-dementia group, four were closely related to neurological and/or HIV disease: dendrite (GO: 0030425) (Figure 2A), ATPase activity coupled to transmembrane movement of ions (GO:0042625) (Figure 2C), ATPase activity coupled to transmembrane movement of ions phosphorylative mechanism (GO:0015662), and cytoskeleton dependent intracellular transport (GO:0030705) (Figure 2E).

Functional annotation results of miRNA target genes were consistent with that of mRNA results, but noteworthy is that the miRNA target genes showed relatively more comprehensive biological processes and cellular components, which could be attributed to the ability of single miRNA to have numerous mRNA targets, therefore further validation might be needed to precise the specificity. Cellular components of miRNA target genes are mainly distributed in cellular (61%) and neuronal related-structures (19%)(Figure 1D), but they display scattering into ion channel, actin cytoskeleton, tight junction, transcription factor complex and chromatin, which concur with our mRNA results. For instance, we found significant dysregulation of genes in microtubule assembly (MAP4 and EML3), microtubule nucleation (TUBGCP6), cytoskeleton movement (SPTBN1) and microtubule stabilization (CALD1). In regards to ion channel genes, we found the upregulation of genes related to ion transport or ion channel, including Ca^2+^, Cl^-^, Na^+^, K^+^, Cu^2+^, Zn^2+^ and glutamine transport. Especially, the genes involved in Ca^2+^, Na^+^ and glutamine transport changed greater than 1.5 fold, the dysregulation of which is known to play a significant role in pathophysiology of neurodegenerative diseases as discussed in subsequent sections. We also found more than 20 genes dysregulated in transcriptional regulation process, and similarly, the biological process of small percentage of miRNA target genes fall into cell adhesion, tissue/embryonic development, learning, and chromatin modification etc. (Figure 1B). Although they were not dominant, they all can play a very important role in neurological degeneration [30-33].

### Quantitative real time RT-PCR corroboration of mRNA and miRNA array dataset

In order to confirm and validate the data obtained by both data sets, we analysed 11 DE genes and 6 DE miRNAs and 1 non-changed miRNA using quantitative real time RT-PCR on the same study sample set.

**Figure 3:**
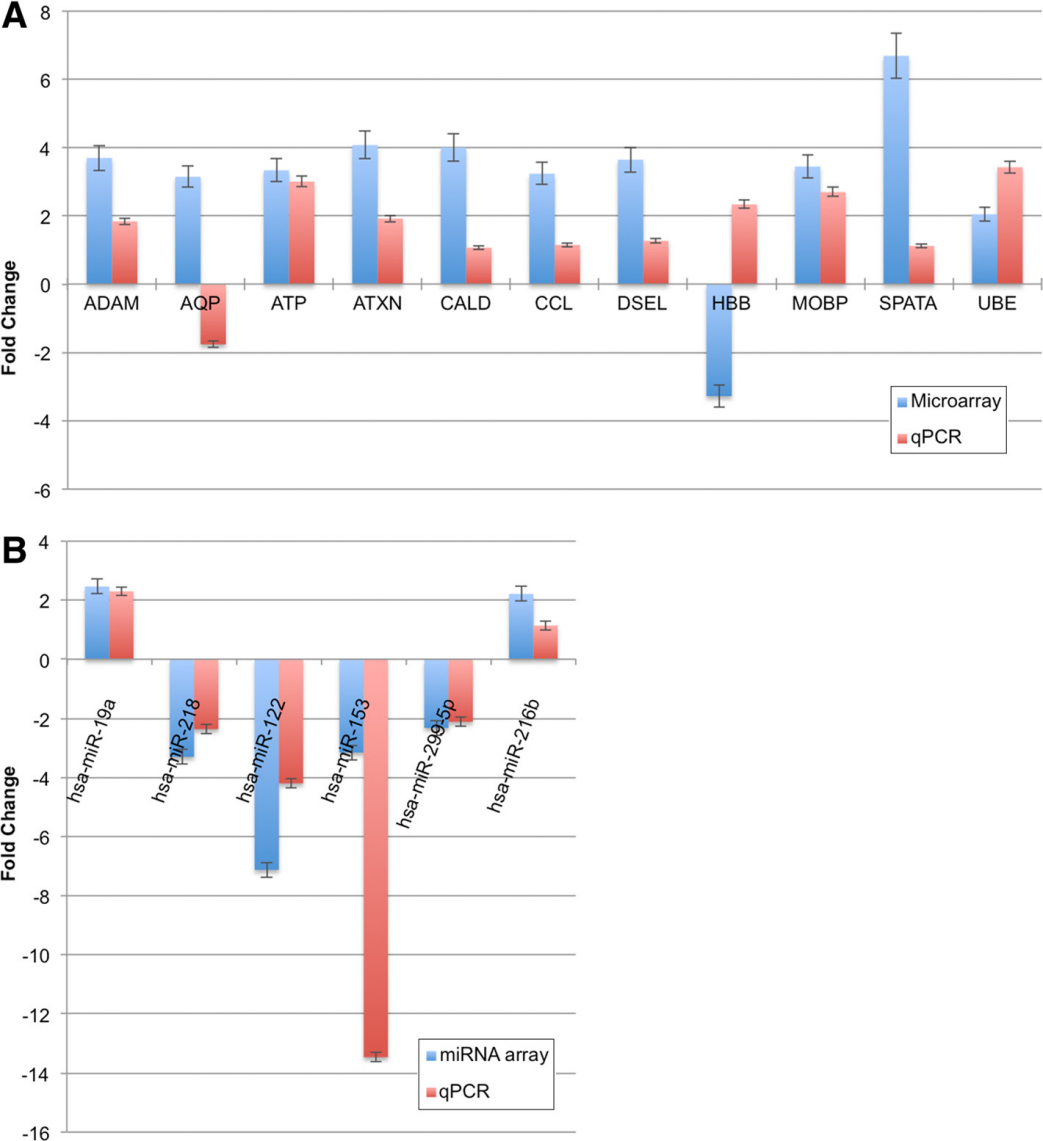
**Validation of mRNA and miRNA profiles using quantitative RT-PCR on the same sample set of microarray study. A**. mRNA profiles validation. **B**. miRNA profiles validation. Data was analysed using the 2^-ΔΔCt^ method and results were plotted as fold differences of relative expression normalized to house keeping gene (GAPDH) and Hs_RNU6B_3 individually. The data have been collected from the same RNA samples from which mRNA and miRNA profiling have been done. All the data presented in this figure have significant P value of <0.05 as calculated by Student's t-test.

For mRNA, the quantitative real time RT-PCR for 9 of the 11 genes was fully consistent with their microarray expression profiles and trends (P<0.05) (Figure 3A). The two genes (AQP and HBB) for which the RT-PCR was not consistent with the microarray were excluded from further analysis. This, in no way, compromises the interpretation of our dataset.

For miRNA, the quantitative RT-PCR for 7 of 7 miRNAs was consistent with the trend seen in microarray analysis (P<0.05) (Figure 3B and Additional file 7), which enhances the confidence and shows the validity of our miRNA data set and their gene targets defined herein.

### Pathway analysis of DE mRNAs and miRNAs

DE gene list and DE miRNA target gene list were uploaded to DAVID to explore functionally relevant pathways.

**Figure 4:**
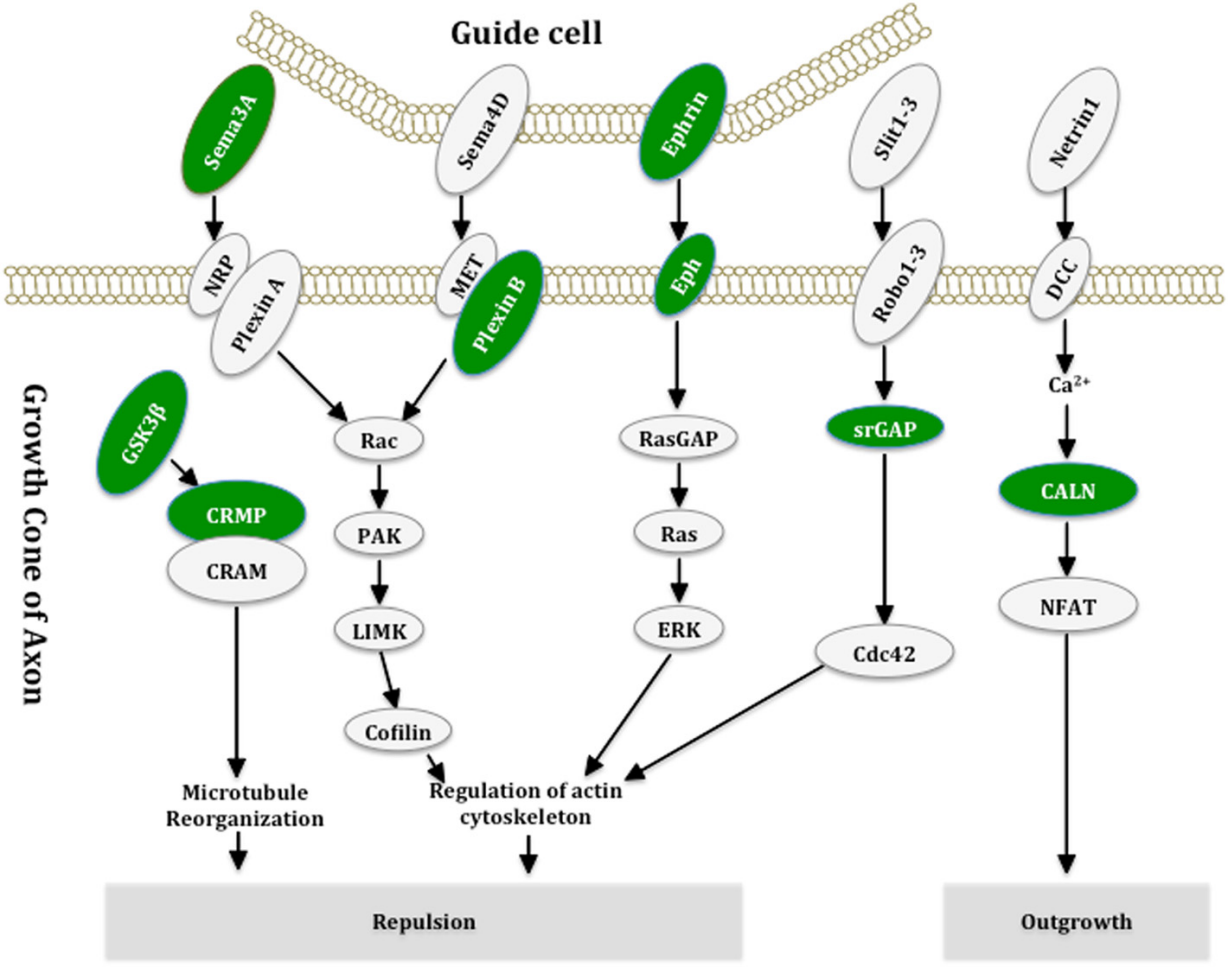
**Significantly down-regulated genes on axon guidance pathway in HAD brains compared to HIV non-dementia brains.** Pathway analysis has revealed the significant involvement of axon guidance molecules and their receptors in HAD brains. The genes, which were found to differentially express between HAD versus non-demented patients in the current study are highlighted in green.

**Figure 5:**
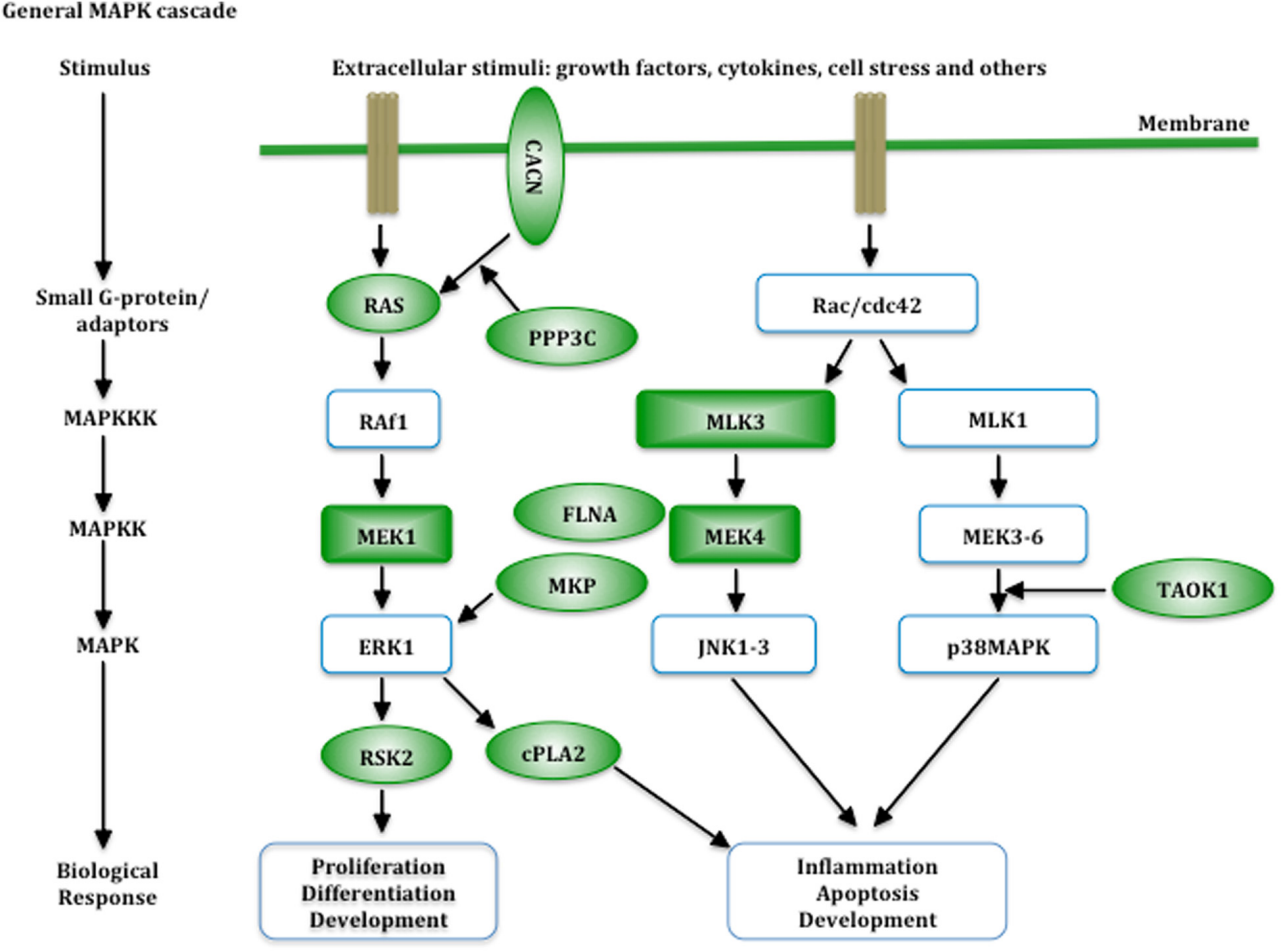
**Significantly down-regulated genes on MAPK pathway in HAD brains compared to HIV non-dementia brains.** Pathway analysis has revealed the significant involvement of MAPK pathway in HAD brains. The genes, which were found to differentially express between HAD versus non-demented patients in the current study are highlighted in green. The rectangles represented members of Mitogen-activated protein kinases family while the ovals are related genes involved in MAPK pathway.

For mRNA, long-term potentiation, axon guidance and signalling pathways (including MAPK signalling pathway, calcium signalling pathway, Jak-STAT signalling pathway and VEGF signalling pathway) stood out significantly. Not surprisingly, we found 7 genes significantly dysregulated in long-term potentiation pathway (CHP, CACNA1C, ITPR1, MAP2K1, PPP1R12A, PPP3CA and RPS6KA1) (Additional file 8). Among them, ITPR1 and PPP3CA down regulated greater than 1.5-fold change. Axon guidance pathway was significantly down regulated in HAD versus non-demented patients with 9 down regulated genes (CHP, EPHA4, EFNB2, SEMA3A, PLXND1, SRGAP1, GSK3B, DPYSL2 and PPP3CA) (Figure 4). Among them, EPHA4 was down regulated >2 fold, while PLXND1, SRGAP1, PPP3CA were down regulated >1.5 fold. There were a total of 27 genes involved in signalling pathway. We found that all the 12/27 genes in the MAPK pathway were down regulated, including the key members of the MAPK signalling pathway, such as MAP2K1, MAP2K4, MAP3K11, RRAS2, RPS6KA1 and TAOK1. Among them, TAOK1 was down regulated greater than 2 fold, while MAP2K4, MAP3K11, PLA2G4A and RRAS2 was dysregulated greater than 1.5 fold (Figure 5). Two key proteins in the MAPK pathway, MAP2K2 (MEK2) and MAP2K4 (JNK), were also further validated by western blotting analysis using 4 samples from HAD and 4 samples from HIV non-dementia group, respectively. Both proteins showed down-regulation in HAD brains, which followed the same trend observed in our microarray analysis. MEK2 was recognized by p-MEK-1/2 antibody at 45 kDa (see Additional file 9). It was expressed slightly weaker in HAD brains as opposed to HIV non-dementia patients (FC=1.1, Additional file 9). Interestingly, this difference was more prominent when severe dementia and non-dementia patients were compared (FC=1.7), which indicates its significance in HAD pathogenesis. MAP2K4 was recognized by JNK1/3 antibody at 46 kDa (Additional file 9). It was downregulated in the HAD brain as well (FC=1.5). There are 11 genes dysregulated in the calcium signalling pathway, 7 in Jak-STAT signalling pathway and 5 in VEGF signalling pathway (there are overlapping genes across different signalling pathways). The details were listed in Additional file 10, Additional file 11 and Additional file 12.

It is worth mentioning that most of the core-enriched genes contributing to each individual gene set significantly enriched in GSEA analysis fell into neurodegenerative disease related pathways, such as tight junction KEGG pathway, neurodegenerative disease pathway, MAPK signalling pathway, axon guidance pathway, and phosphorylative mechanisms/signalling pathway (for details see in Figure 2B, D and F). These results are consistent with our previous observations [34] and functional annotation analysis, therefore further confirmed the significant involvement of these pathways in HAD pathogenesis.

For miRNA, a number of significantly involved pathways (P<0.05) were revealed, including signalling pathways, adhesion/junction, axon guidance, depression/potentiation, apoptosis/cell cycle, inflammation-related pathways, ubiquitin mediated proteolysis, and regulation of actin cytoskeleton (Additional file 13). Notable was that several pathways were targeted by more than 5 DE miRNAs. For instance, the wnt-signalling pathway was targeted by 10 DE miRNAs, the axon guidance pathway and endocytosis pathway by 9 DE miRNAs, insulin signalling pathway, long-term potentiation pathway and focal adhesion pathway by 7 DE miRNAs. Interestingly, the DE hsa-miR-19a targeted all 6 pathways listed above, whereas the DE hsa-miR-137, hsa-miR-153 and hsa-miR-218 targeted 5 pathways, and the DE hsa-miR-323 and hsa-miR-495 targeted 4 pathways (Additional file 13). Following the incorporation of mRNA pathway and GSEA analysis results, this is a highly comprehensive dataset in the context of neurodegeneration and pathogenesis. In addition, we also found several cancer-related pathways significant as well, which were consistent with the fact that viruses can trigger or be co-factor of cancers and that a number of cancer genes are pro-inflammation, a scenario also seen in HIV infection and in neurodegenerative process, where HIV initiates cascade of pro-inflammatory mediators (cytokines, chemokines and adhesion molecules) and upregulation of their respective genes during infection [35].

### Correlation between expression levels of DE miRNAs and DE mRNAs

**Figure 6:**
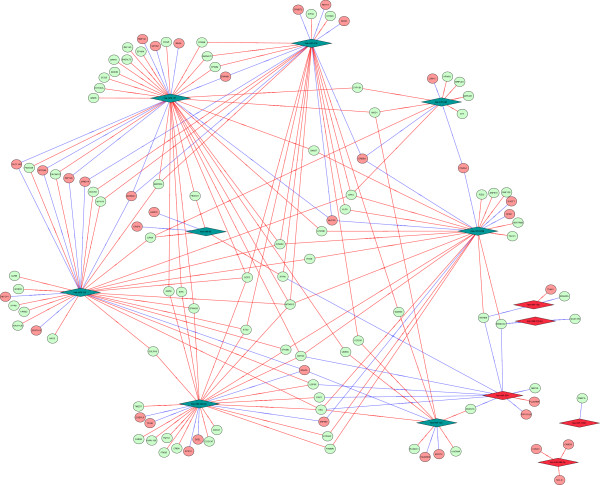
**miRNA-mRNA correlation network.** miRNA-mRNA correlation network discovered by SA-BNs, rendered in cytoscape [39]. In this network, the miRNAs are demonstrated by diamond shape while mRNAs with circles. Upregulated miRNA are in red, while downregulated miRNA are in green. Upregulated mRNAs are in pink, while downregulated are in light green. The positive correlated miRNAs and mRNAs are connected by red line, while the negative correlations by blue lines.

**Figure 7:**
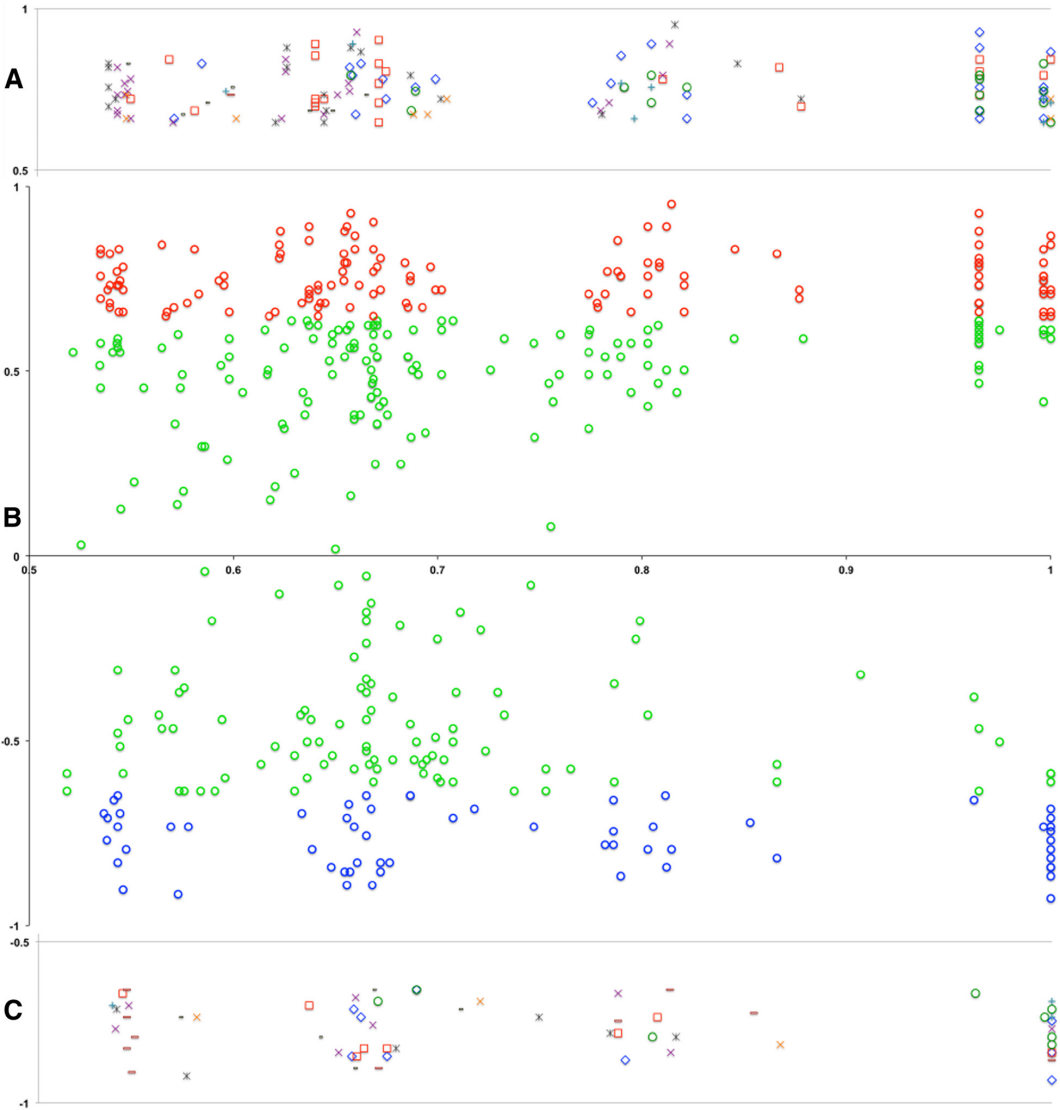
**Pearson's correlation of miRNA-mRNA pairs vs. significant confidences of their interactions discovered by SA-BNs. A**: Depiction of positive interactions from each miRNA. **B**: Pearson's correlation of miRNA-mRNA pairs vs. significant confidences of their interactions discovered by SA-BNs. The X-axis shows the confidence of interactions, ranging from 0 (the least confident) to 1 (the most confident), while only the statistical significant scale remains (P<0.05). The Y-axis shows the sample correlations of identified miRNA-mRNA pairs. The red and blue dots are miRNA-mRNA pairs, which are correlated at the significant level (P<0.05). It shows that the identified interactions are largely correlated, either negatively or positively, suggesting direct interactions and indirect interactions correspondingly. **C**: Depiction of negative interactions from each miRNA ◊miR137 □miR153 ^O^miR218 ×miR299-5p *miR376a ^X^miR122 ^+^miR592 ^–^miR584 ^-^Others.

We evaluated the significance levels of all possible correlations between DE mRNAs and miRNAs using SA-BNs [36]. We found 438 interactions with high confidence in total. Among them, 195 were statistically significant (P<0.05), including 13 miRNA and 116 mRNA, whose expression levels correlated with each other according to Pearson’s correlation (P<0.05) (Figure 6). The Pearson's correlation of miRNA-mRNA pairs vs. significant confidence of interaction discovered by SA-BNs has been shown in Figure 7. Interestingly, most of them correlated positively although miRNAs were downregulated. Among them, hsa-miR-137, hsa-miR-153, hsa-miR-299-5p, hsa-miR-218 and hsa-miR-376a were outstanding due to their functional correlation with numerous genes. It is interesting to see that most of the miRNA are down regulated in HAD versus HIV non-demented brains, and positively correlated with their mRNA target, which is supported by previous findings [37,38].

**Figure 8:**
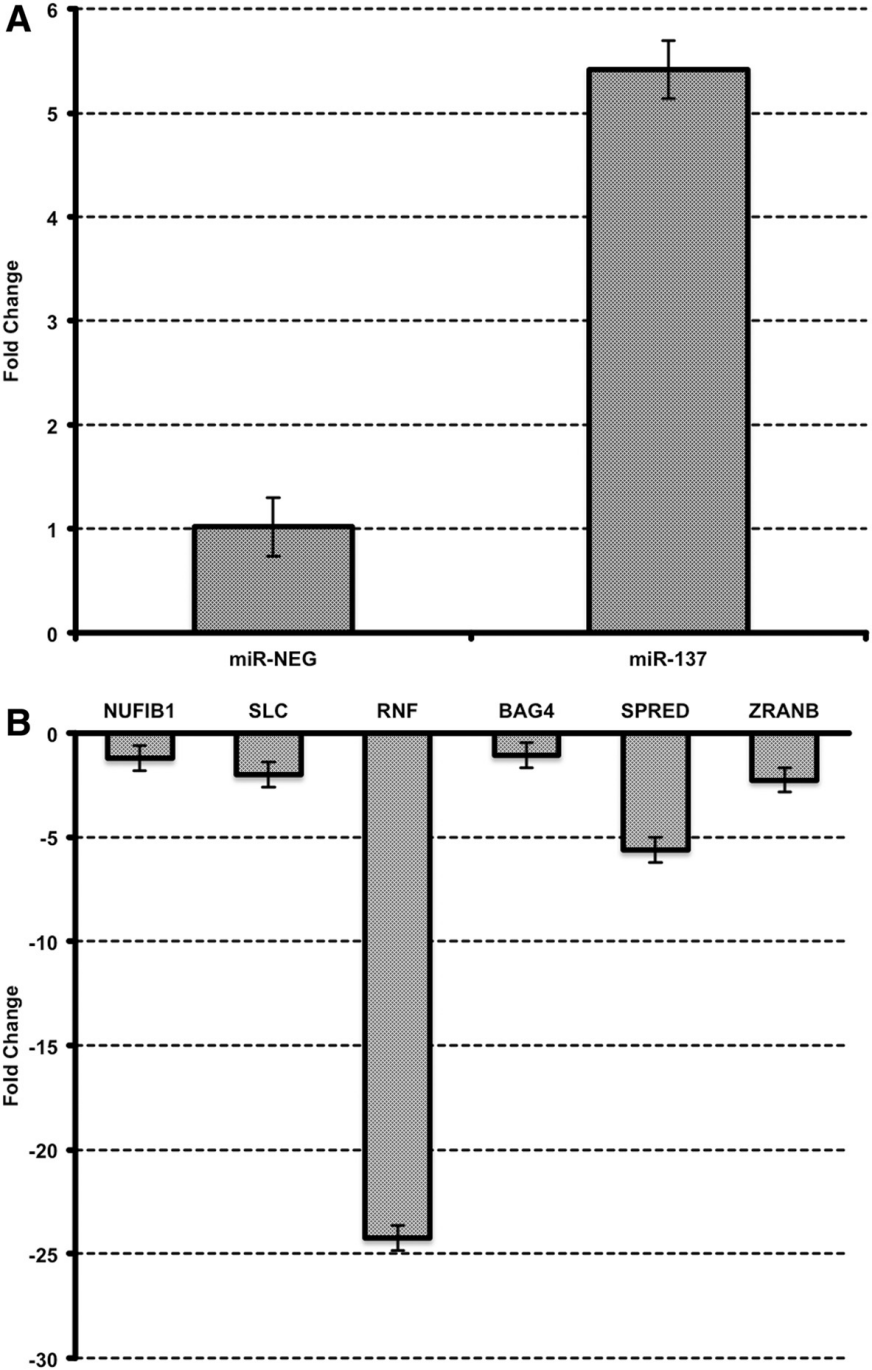
**Effect of the transfection of microRNA mimic of miR-137. A**. Transfection of neuroblasts showing basal expression of miR-137, based on which our experiments on miR-137 target genes were carried out. **B**. Column chart of fold changes of expression values (qRT-PCR) between SH-SY5Y transfected with miR-137 mimic or miRNA mimic negative control. Values are based on fold change calculated from 2^-ΔΔCt^ method. All the columns have significant P value of <0.05 as calculated by Student's t-test.

To validate this correlation further, miRNA mimic of miR-137 and negative control treatments were carried out. That led a significant decrease in expression levels of NUFIB1, SLC, RNF, BAG4, SPRED, ZRANB at 24 h after transfection (Figure 8), which are all the genes negatively regulated by miR137 according to the correlation network we found. This result added extra confidence to our correlation network.

## Discussion

This is the first joint study of whole-genome mRNA and miRNA profiling using individual human brain RNA from autopsies of HAD and HIV non-dementia patients. In this study, we initially compared mRNA and miRNA data at the clustering, gene ontology (including cellular components and biological processes), and pathway levels. Following that, SA-BNs correlating miRNAs and mRNAs by their expression levels were performed to validate the accurate prediction of genes potentially targeted by dysregulated miRNAs.

The clustering and gene ontology results showed excellent functional concordance between mRNA and miRNA, demonstrating the significant involvement of neuronal cellular components and biological processes such as: signal transduction, transcriptional regulation, metabolism, response to stimuli, cell cycle/apoptosis, protein modification, neuronal processes and ion transport, respectively. This intrinsic functional consistency between miRNA and mRNA has given an extra power to our findings in relation to understanding the genomic basis of HIV neuropathogenesis and HIV-mediated neurodegeneration. Moreover, the DE miRNAs were more robust than their mRNA counterparts in providing a comprehensive snapshot of cellular components and biological processes involved in neuropathology and neurodegeneration. Compared to DE miRNAs, DE mRNAs could only predict elemental functional pathways and processes related to neuropathology. DE miRNAs revealed the participation of additional cellular components (eg. ion channel, actin cytoskeleton, tight junction) and biological processes (cell adhesion, tissue/embryonic development, learning), which also concurs with biological processes of mRNA. Interestingly, these findings are consistent with study, which has been done using CSF of HIVE patients [40]. The most plausible explanation for the comprehensiveness of miRNA coverage as compared to their mRNA counterpart is that a single miRNA or the miRNAs belonging to the same family in the cluster can target several hundred genes within a biological process or pathway. Therefore, it is not surprising that miRNA gives broader information compared to mRNA. Secondly, there are many other gene regulational mechanisms apart from miRNA and together they can compensate the effect of miRNA dysregulation to some degree, consequently the miRNA regulation effect can only be seen after certain stage of disease progression when other mechanisms are not sufficient any more [41].

Pathway analysis of the joint mRNA and miRNA results provided the first *in vivo* evidence of significant involvement of axon guidance pathway and its downstream signalling pathways (such as MAPK signalling pathway and Ca^2+^ signalling pathway, etc.) on both transcriptional level and regulation level. Axon guidance pledges precise path finding and defines their termination zones and synaptic partners [42,43], which is fundamental to neuronal development and networks. In addition, misrouted fibers have been shown in AD and PD’s brains [44]. Furthermore, it is well known that HIV envelope glycoprotein (GP120) can cause axonal degeneration [45] and recently axon damage has been claimed as a key predictor of outcome in multiple neurological disorders, including HAD [46]. Axon guidance pathway contains four prominent families of ligands (ephrin, netrin, semaphorin and slit proteins), their receptors and downstream signalling proteins. The role of axon guidance pathway molecules in the maintenance and plasticity of neural circuits has been reported [47]. Moreover, the variations (single-nucleotide polymorphisms) in axon guidance pathway genes have been reported to predict PD outcomes [48]. Significantly, 9 of our DE miRNAs have been found targeting this pathway according to TargetScan results; and more importantly these results support previous observations with 3 out of 4 ligands/receptors being dysregulated in our mRNA studies, including ephrin(EPH) receptor A4 (EPHA4), netrin G2 (NTNG2), and semaphoring 3A (SEMA3A), strongly suggesting the impairment of axon guidance pathway in HAD brains *in vivo*.

Moreover, our results highlight axon guidance downstream signalling pathways, which allow precise patterns of connectivity within the CNS. For instance, the MAPK pathway comes out significant in both our mRNA and miRNA profiling. Studies have shown that the activation of MAPK is necessary for axon guidance [49], and it contributes to netrin signalling in axon guidance [50]. Besides, netrin-dependent axon outgrowth and orientation can be antagonized by inhibition of ERK-1/2 [50]. The role of MAPK pathway in HIV infection has also been well documented. For instance, it has been reported that the MAPK pathway plays a crucial role in HIV-1 replication and virulence [51] and is one of the transcriptional signatures in HIV+ long-term non-progressors [52]. In addition, the binding of HIV-1 GP120 to CD4 receptors on T cells can activate the MAPK pathway and induce transcription of cytokine and chemokine genes [53]. Interestingly, MAPK pathway was targeted by 3 DE miRNAs and it includes 11 of our DE genes, such as RPS6KA1, FLNA, RRAS2, and MAP2K4 etc., each of which play an important role in MAPK signalling. MAPK signalling cross-talks with the Jak-STAT signalling pathway at multiple levels. In mammals, the Jak-STAT signalling pathway is the principal signalling mechanism for cytokines and growth factors and therefore plays a key role in cell proliferation, differentiation, cell migration and apoptosis. Supporting this relationship, Jak-STAT was highlighted in our analysis as well. We found 7 genes significantly dysregulated in that category (including STAT) and it was targeted by DE miR-19a. Dysregulation of Jak signalling might result in inflammation [54], which is commonly accepted as an important mediator in the pathogenesis of neurodegeneration. VEGF signalling pathway is another significant pathway revealed by our results, and it closely links to MAPK signalling pathway as well. Via activating MAPK signalling pathway, VEGF can exert direct effect on multiple types of neuronal cells, including neurons, astrocytes, and microglias [55]. VEGF also has been reported to be involved in vascular permeability [56] and several studies have shown the potential utility of inhibiting VEGF signalling pathway in reducing BBB disruption [57,58]. Besides, Ca^2+^ can mediate guidance receptor signalling *in vitro*[59] and change in Ca^2+^ concentration can signal growth cone turning [60]. Equivalently, guidance cues can also trigger Ca^2+^ influx and alteration in Ca^2+^ concentration or slope its gradient, thereby influencing the outcome of growth cone behavior (attraction or repulsion) [61,62]. Our studies have demonstrated several genes related to Ca^2+^ transport/signalling dysregulated, including ATP2B4, which play a critical role in intracellular calcium homeostasis.

In addition, endocytosis is another critical aspect of guidance receptor activation and signalling [59]. Nine of our DE miRNAs were found targeting this pathway and several key genes were found dysregulated. Efficient cell detachment needs the endocytosis of the ephrin-Eph complex, or even bidirectional endocytosis for ephrinB-EphB-induced repulsive guidance. In addition, endocytosis also plays a role in regulating the senstivity of the growth cone correspondent to a repulsive cue. Again, 9 of 68 of our DE miRNAs targeted endocytosis pathway. Our mRNA study also revealed dysregulation of Ras related protein (RAB4B) and EHD protein (EHD4), which are important components of endocytosis pathway. We also found ADAM22 dysregulated, whose family member ADAM10 has been reported to play a role in converting initial adhesive interaction into repulsion and therefore providing an effective strategy for axon detachment and attenuation of signalling [63,64].

Further, our miRNA and mRNA Bayesian correlation analysis has provided an unambiguous snapshot of miRNA and mRNA functional interactions and their biological significance. Sophisticated Bayesian Structure learning approach defines miRNA-mRNA interactions based on their relative expression of all of these molecules in each condition. This network-based approach identified these key interactions with very high confidence. These interactions define the network topography that is provided by Bayesian statistics and is substantially more rigorous than individual correlations that can be defined conventionally. These relationships, therefore, are more likely to be meaningful at the system level compared to reporter assay. We identified 195 positive and negative statistically significant correlations (P<0.05) between our DE mRNAs and miRNAs. It strengthens the correlation and shows the credibility of Bayesian analysis we have performed for establishing this intrinsic functional relationship between mRNA and miRNA. Among them, two thirds of functional correlations are from 5 DE miRNAs: miR-137, 153, 218, 376a and 299-5p. They all changed greater than 2 fold. Since all of them have been reported to be involved in classical neurodegeneration, for instance, miR-376a has been reported to mutate in Huntington’s disease (HD) brains [65]; miR-299-5p has been reported to be dysregulated in multiple sclerosis (MS) brains [66] and more interestingly, all of them have been shown to be dysregulated in the AD brains [67]. In particular, miR-137, 153 and 218, which can target more than 5 neurodegeneration-related pathways, implying their functional relevance to the observations noted in this study. The miR-137 has been shown to be enriched in neurons, especially within the dentate gyrus and the molecular layer of adult hippocampus [68] and studies have shown that it plays an important role in modulating neuronal cell proliferation and differentiation [68-71]. Moreover, it has been shown to be genetically associated with schizophrenia [48]. Recently, it has been shown dysregulated in the CSF of HIVE patients [40], which is consistent with the expression of miRNA-137 in the frontal cortex in our study in HAD patients. In addition, the miR-218 is enriched in hippocampus [72] and altered miR-218 expression has been reported in HD and MS brains [66,73]. The miR-153 has been shown to play important role in AD and PD pathogenesis [74,75]. It can downregulate the expression of APP protein *in vivo*, suggesting its possible role in AD pathogenesis [74]. Moreover, it can regulate α-synuclein, which is the primary structural components of Lewy bodies, indicating its role in PD pathophysiological process [75]. The genes that correlated to are all involved in several significant pathways discussed above. In particular, SPRED1, MAP2K4 and DIRAS2, they all correlated with 3 out of 3 miRNAs (miR-137, 153 and 218) and they all appear to be involved in MAPK signalling pathway, which strongly indicate the participation of MAPK pathway in HAD pathogenesis and is consistent with our previous proteomic studies [34].

Although our study is the first comprehensive parallel genome-wide mRNA and miRNA profiling of HIV infected human brains, there are still limitations: 1. In future, a bigger sample size, blood and CSF samples will be needed to further validate these findings, and confirm the clinical value of this findings; 2. These findings are based on the whole human brain cortex rather than specific cell types (eg. neurons) due to lack of plausible and effective methodologies for perfect cell separation. Although Laser Capture Microdissection (LCM) is currently used in studying cell types, there are significant limitations in profiling single cell type [76]: A. Due to the minimum laser spot size (7.5 μm), it poses a limit to the precision of single cell micro-dissection without contaminating fragments of adjacent cells. B. Due to its suboptimal optical resolution on uncovered sections, it will compromise cell borders distinction and result in cytoplasmic compartment loss, which is crucial for our mRNA analysis. Although immunohistochemical (IHC) staining will circumvent this problem to some degree, it is impossible to recover cytoplasmic compartment precisely without contamination. Moreover, IHC procedures, tissue fixation and LCM capturing of cells dramatically affect RNA yield. C. Sectioning will generate large number of attached fragments, which might alter expression profiles greatly. In addition, due to the lack of the cytoplasm or even the nucleus, the genomic information will be considerably compromised.

Overall, our study provides a strong foundation and durable framework for systematic large-scale studies on HIV-infected adult brain to define functional genomic phenotypes of neurodegenerative diseases and functional networks between miRNA and mRNA, which may lead to the development of new generation of prognostic and diagnostic markers and therapeutic intervention strategies for viral and non-viral neurodegenerative diseases.

## Conclusions

This study is the first report on whole-genome joint mRNA and miRNA profile analysis from individual native brain tissue from HIV+ patients with and without dementia and it underscores the important role of intrinsic functional correlation between mRNA-miRNA, which is closely tied to HIV-mediated neurodegeneration. Through mRNA and miRNA joint profiling this study has provided the first thorough *in vivo* evidence on the genomic basis of HIV-mediated neurodegeneration and its correlation with miRNA. This provided a firm support to intrinsic functional relationship that exists between mRNA and miRNA in guiding neurodegeneration in HIV-infected brains. From the concordance between miRNA and mRNA, it demonstrates the significant involvement of axon guidance and its downstream signalling pathways in HIV-mediated neurodegeneration and development of HAD. Most importantly, the most significant dysregulated and highly biological relevant 3 miRNAs identified here, miR-137, 153 and 218, cumulatively targeted the axon guidance pathway as well as its downstream signalling pathways, which may find potential use as diagnostic/prognostic biomarkers and for developing new generation of therapeutic intervention strategies for HIV-associated and possibly other neurodegenerative diseases.

## Methods

### Brain tissue collection

Brain tissue samples were obtained from HIV-1-infected patients with or without dementia through the National Neuro-AIDS Tissue Consortium (NNTC, Request # R203) and the Westmead Hospital, Sydney, Australia (Reference No: 5465). Samples were collected at post-mortem with short delay. The autopsied brain tissue was snap frozen in liquid nitrogen and stored at −80°C until required for use. Frontal cortex of 10 male patients with HAD and 8 without were used for this study due to its importance in motor impairment and involvement in other neurodegenerative disorders, such as: AD [77,78]. The average age for HAD and non-HAD patients was 51.88 ± 9.45 and 43.57 ± 14.77, respectively, (P =0.23). Clinical profiles of all patients are shown in Additional 14. In order to make sure the quality of RNA, samples’ post mortem intervals were less than 48 hours have been selected for the study. Among them, 5 samples from HAD and HIV non-dementia group, respectively, have been randomly chosen for miRNA study. This study was conducted according to the principles expressed in the Declaration of Helsinki. Use of samples in this study was approved by the Institutional Review Board and the Ethics Committee of the NNTC Allocations, the University of Sydney and the Westmead Hospital individually. The family members of the patients gave written, informed consent for the use of autopsied brain tissue. For the diagnostic criteria for HAD, the criteria defined by the American Academy of Neurology 1991 were used [79].

### RNA isolation and mRNA and miRNA profiling

Total RNA was extracted from 30 mg of brain cortex tissue. Tissue samples were homogenised using a high-speed agitation polytron belnder (Kinematica, Luzern, Switzerland) in the presence of RNA lysis buffer (Qiagen miRNeasy Mini kit- Qiagen, USA). The RNA was isolated and purified with a miRNeasy Mini kit with DNAse I digestion on the column (Qiagen, USA) according to the manufacturer’s protocol. The quality and quantitiy of the RNA preparations was assessed using an Agilent 2100 Bioanalyser (Agilent Technologies, USA). RNA integrity scores were>7 for all the samples analyzed. FirstChoice Human Brain Reference commercially available RNA (Ambion, Inc., USA) was used as a control RNA for the microarray analysis.

For mRNA profiling, cRNA amplification and labeling with biotin were performed using Illumina TotalPrep RNA amplification kit according to the manufacturer’s directions (Ambion, USA) with 500ng total RNA as input material. cRNA yields were quantified using Agilent 2100 Bioanalyser. Gene expression analysis was performed using the Sentrix Human-6 v2 Expression BeadChip (Illumina, USA), and BeadStation system from Illumina as per manufacturer’s instructions. The Human-6 v2 Expression BeadChip allows genome-wide expression profiling of more than 48,000 gene transcripts and known alternative splice variants from the RefSeq database. For miRNA profiling, 1000ng total RNA was labelled with FlashTag Biotin HSR RNA Labeling Kit (Genisphere Inc. USA) and analzed using Affymetrix GeneChip miRNA Array (Genisphere Inc. USA), which contains 1105 *Homo sapiens* miRNAs.

Gene expression data analysis was performed using GenomeStudio version 3 (Illumina Inc., Singapore). The gene expression data was normalised using the cubic spline function, the genes were selected if the detection P<0.01 (indicating the transcript was detected) in at least one group. All samples were coded and analyzed blindly to avoid any bias. The differential gene expression analysis was performed using Illumina custom error model with false discovery rate correction implemented in GenomeStudio [80]. Genes, whose DiffScore >13 or <−13 (corresponding to significance level of P<0.05), were considered statistically significant. miRNA data analysis was carried out using GeneSpring 11.0 (Agilent Technologies, USA). The data was normalised by expression percentile and analysed using Mann–Whitney unpaired test. P<0.05 were considered statistically significant.

### Real time quantitative RT-PCR validation of mRNA and miRNA

The data for mRNA and miRNA were selectively corroborated with real-time PCR to ascertain their expression trends. For mRNA, 5ng total RNA was reverse transcribed using oligo d(T) and Superscript III followed by RNase H treatment (Invitrogen Life Technologies), per manufacturer’s instructions. PCR primers (Additional file 15) were designed for all the 11 genes selected on the basis of the microarray data (Additional file 1) as well as for the control genes (GAPDH: glyceraldehyde-3-phosphate dehydrogenase), using the online software Primer 3 (http://frodo.wi.mit.edu/primer3/). All primer pairs were optimized to ensure the specific amplification and the absence of any primer dimer. Quantitative PCR standard curves were set up for all. The cDNA was then subjected to real time quantitative PCR with defined primers and SYBR Green (Invitrogen Life Technologies) using Mx3000p Stratagene real-time thermal cycler (Stratagene, La Jolla, CA, USA). The data were analysed using the MxPro™ QPCR software version 4.0.1 (Stratagene, La Jolla, CA, USA). For miRNA, expression levels of six DE miRNAs (miR-153, 218, 19a, 216b, 122, 299-5p and 280) were validated by quantitative real time RT-PCR using the Qiagen miScript PCR system (Qiagen, Valencia, CA, USA) according to the manufacture’s protocol. Hs_RNU6B_3 was used as the endogenous control to normalize the data. All the experiements were performed in duplicate and relative expression levels of these mRNAs/miRNAs were determined by the 2^-ΔΔCt^ method. The data then were further analysed by Student t-test to check the statistical significance between HAD and HIV non-dementia patients brains (P<0.05).

### Transfection of microRNA mimic

SH-SY5Y cultures were maintained as confluent monolayers at 37°C with 5% CO_2_ and 90% humidity in SH-SY5Y media (DMEM with 10% (vol/vol) foetal calf serum, 20 mM HEPES, 2 mM L-glutamine). For differentiation cells were seeded at 4 × 10^4^ cells/cm^2^ and treated with all-trans retinoic acid (ATRA) media (SH-SY5Y media with 10uM ATRA) for five days, followed by treatment in brain-derived neurotrophic factor (BDNF) media for three days (SH-SY5Y media without foetal calf serum and with 50 ng/ml BDNF) [81]. Cells were then harvested and nucleofected using Amaxa Nucleofector Kit V (Lonza) according to manufacturer’s instructions. Each nucleofection contained 4 × 10^6^ cells and 0.1 nmol miR-137 (T:[Phos]UUAUUGCUUAAGAAUACGCGUAG B:[mA][mC]GCGUAUUCUUAAGCAAUAG[dT] [dT]) or mirVanaTM miRNA mimic Negative Control #1, with experiments performed in duplicate. Nucleofected cells were seeded at 5 × 10^4^/cm^2^ in BDNF media and grown for 24 hrs before being harvested with TRIzol and RNA isolated as described [82].

### Functional validation of proteins using western blot

Western blot was employed to validate part of the microarray data. 4 HAD patients and 4 HIV non-dementia patients’ brain samples were used for validation of the microarray study by western blot analysis. Total cellular proteins were extracted as described before [34]. 40 μg proteins were separated by 12% SDS-polyacrylamide gels and then transferred to PVDF membranes (Millipore, USA) or nitrocellulose membranes (Amersham, USA) using Bio-Rad apparatus (Bio-Rad, USA). Membranes were blocked in 5% skim milk powder or 5% BSA in tris-buffered saline (TBS) (20 mM Tris and 0.9% NaCl, pH 7.4) for 1 hour at room temperature. Following that, they were incubated for 2 hours at room temperature with each of the following primary antibodies: Rabbit anti-MEK2 (1:2000) and JNK1 (1:1000) (Santa Cruz. USA). Mouse anti-Actin (1:6000, DAKO, USA) was used as control antibody. Membranes were washed four times with TTBS (TBS with 0.05% Tween20) and then incubated for 1 hour with anti-rabbit or anti-mouse HRP-conjugated secondary antibody (Dako, USA; 1:6000) followed by chemiluminescence ECL detection (GE, USA) and exposure to autoradiography film (Kodak, France). Films were scanned with HP scanjet8200 (HP, USA) and the images were collected and analysed using ImageJ software (http://rsbweb.nih.gov/ij/). Statistically significant differences between patients were estimated with the Student t-test (P<0.05).

### Data analysis

For mRNA, gene ontology analysis has been carried out using DAVID and GSEA. Illumina ID of differential expressed genes was uploaded to the DAVID database and the analysis was performed using the algorithm within the softwares. With GSEA, the whole genome (27455 genes) with expression value were uploaded to the software and compared with catalog C5 gene ontology gene sets in MsigDB [29], which contains 233 GO cellular component gene sets, 825 GO biological process gene sets, 396 GO molecular function gene sets. For miRNA, TargetScan was used to find the global target of DE miRNAs, which were dysregulated by at least two fold and the target gene list was uploaded to DAVID as well. mRNA and miRNA correlation analysis has been performed using SA-BNs.

## Competing interests

The author(s) declare that they have no competing interests.

## Authors’ contributions

L Zhou fully performed the work, analyzed data and drafted the paper. GM Pupo assisted with RNA amplification and microarray hybridization. SL Tran and H Rizos provided full assistance with the WB validation analysis. B Wang assisted with real time RT-PCR and data analysis. R Rua participated in the RNA extraction and quantitation. NK Saksena conceived, designed, supervised and coordinated, along with providing assistance with drafting the manuscript; P Gupta and R Rahme provided assistance with miRNA extraction and miRNA Affymetrix array. M Cairns and B Liu assisted with bioinformatic and miRNA Affymetrix analyses. A Carroll provided assistance with miRNA knockdown experiment. Part of the work on miRNA reported herein was supported by the funding from the Judith Mason Foundation to NKS. All authors read and approved the final manuscript.

## Supplementary Material

Additional file 1 **Table S1.** DE gene list. Click here for file

Additional file 2 **Figure S1.** Hierarchical clustering analysis of global mRNA and miRNA profiles obtained from the frontal cortex tissue at autopsy of HIV patients with and without dementia. Pearson correlation algorithm was chosen to evaluate and visualize the mRNA and miRNA expression patterns using GenomeStudio v3 and GeneSpring respectively. **A.** mRNA clustering. HAD samples are highlighted with (*red square*), and HIV negative control samples are highlighted with (*sky-blue square*) while HIV positive non-dementia patient samples are highlighted with (*green square*). **B** miRNA clustering. Click here for file

Additional file 3 **Table S2.** DE miRNA list. Click here for file

Additional file 4 **Figure S2.** Target of hsa-miR-137. Click here for file

Additional file 5 **Table S3.** Low and high stringency G-seed search for miR-137, miR-153 and miR-218 targets. Click here for file

Additional file 6 **Table S4:** Gene sets significantly enriched by GSEA analysis. Click here for file

Additional file 7 **Table S5.** Validation of non-changed miRNA. Click here for file

Additional file 8 **Figure S3.** Significantly dysregulated genes on long term potentiation pathway in HAD brains compared to HIV non-dementia brains. Click here for file

Additional file 9 **Figure S4.** Western blot validation of MAP2K2 and MAP2K4. MAP2K2 and MAP2K4 encoding proteins MEK2 (A) and JNK (B) with relative molecular weight 45 and 46kD respectively were separated on 12% SDSpolyacrylamide gel and blotting with specific antibodies against them. Semiquantitative analysis has been carried out by comparing the relative protein level (standardized by Actin (C)) in HAD and HIV non-dementia patients. The quantification result demonstrated the trend similar to the one observed in microarray and qPCR for HAD patients when compared to HIV non-dementia patients. Click here for file

Additional file 10 **Figure S5.** Significantly dysregulated genes on calcium signalling pathway in HAD brains compared to HIV non-dementia brains. Click here for file

Additional file 11 **Figure S6.** Significantly dysregulated genes on Jak-STAT signalling pathway in HAD brains compared to HIV non-dementia brains. Click here for file

Additional file 12 **Figure S7.** Significantly dysregulated genes on VEGF signalling pathway in HAD brains compared to HIV non-dementia brains. Click here for file

Additional file 13 **Table S6.** DE miRNAs and their target pathways. Click here for file

Additional file 14 **Table S7.** Patient details. Click here for file

Additional file 15 **Table S8.** qPCR primers. Click here for file
