# Supplementary figures and images for: A parallel genome-wide mRNA and microRNA profiling of the frontal cortex of HIV patients with and without HIV-associated dementia shows the role of axon guidance and downstream pathways in HIV-mediated neurodegeneration
Source: BMC Genomics. 2012 Nov 28;13:677. doi: 10.1186/1471-2164-13-677 (PMC3560210; doi:10.1186/1471-2164-13-677)

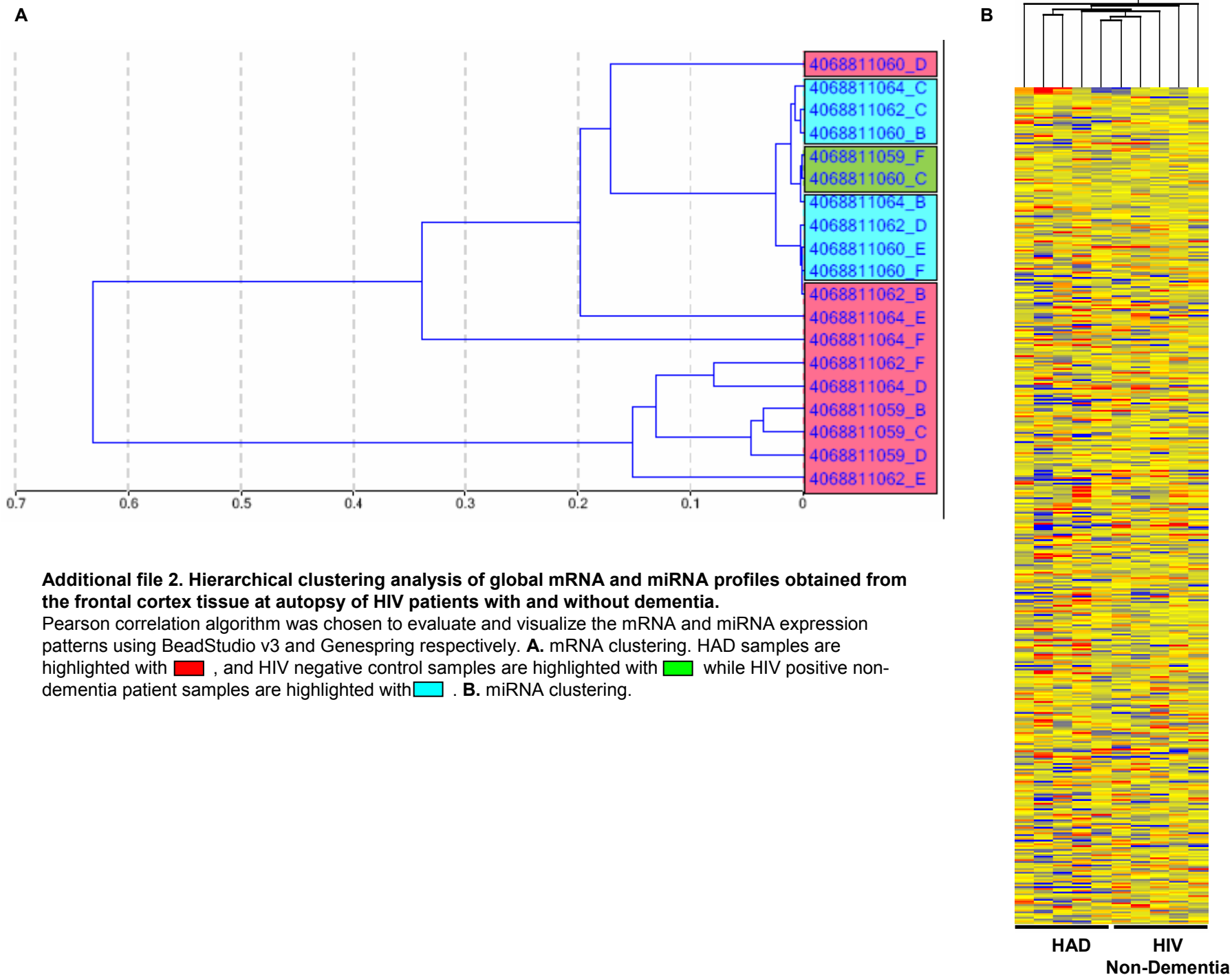

Supplement: Additional file 2 — Figure S1. Hierarchical clustering analysis of global mRNA and miRNA profiles obtained from the frontal cortex tissue at autopsy of HIV patients with and without dementia. Pearson correlation algorithm was chosen to evaluate and visualize the mRNA and miRNA expression patterns using GenomeStudio v3 and GeneSpring respectively. A. mRNA clustering. HAD samples are highlighted with (red square), and HIV negative control samples are highlighted with (sky-blue square) while HIV positive non-dementia patient samples are highlighted with (green square). B miRNA clustering. [file 1471-2164-13-677-S2.pdf]

## Slide 1
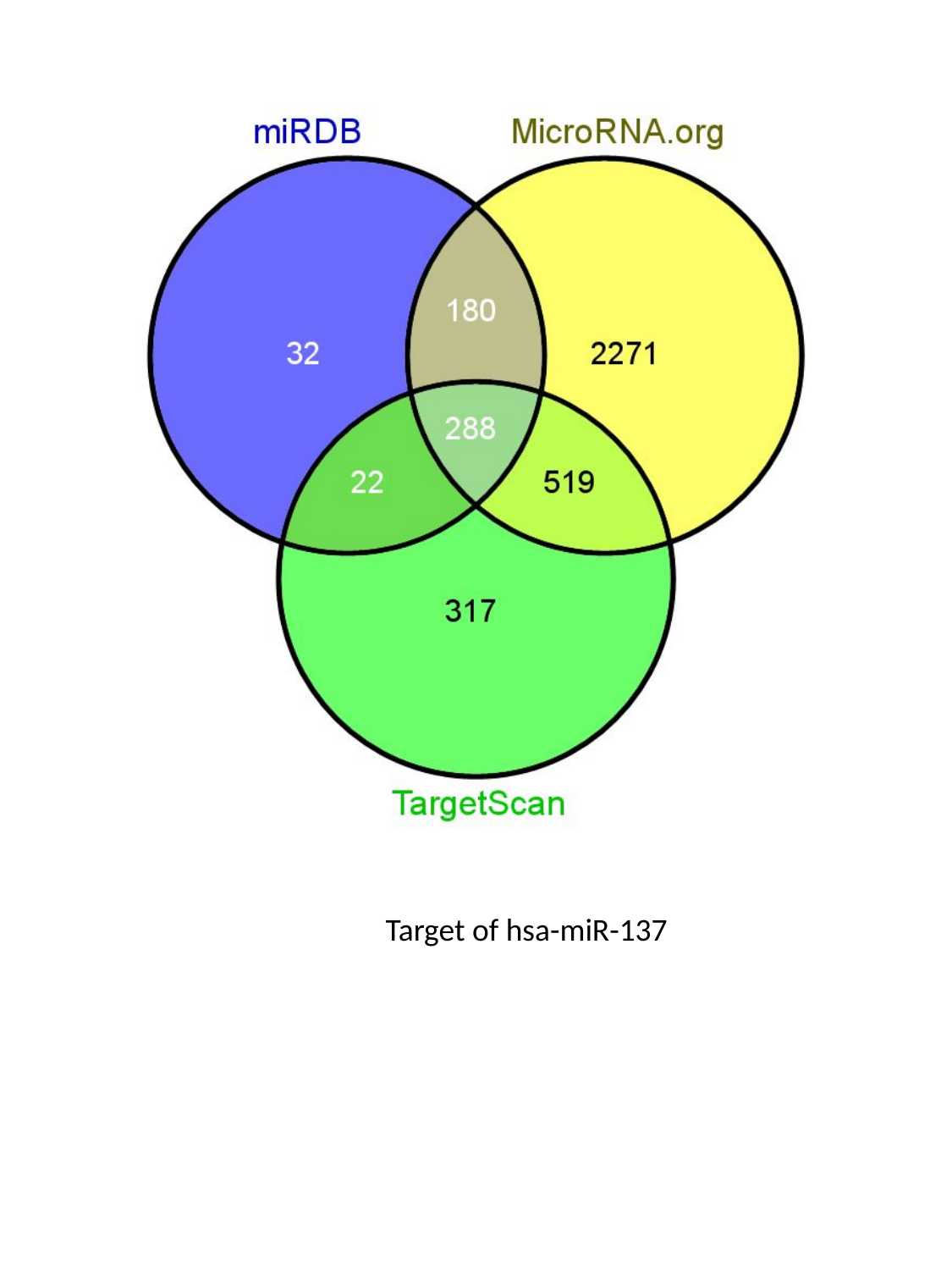

Target of hsa-miR-137

## Slide 2
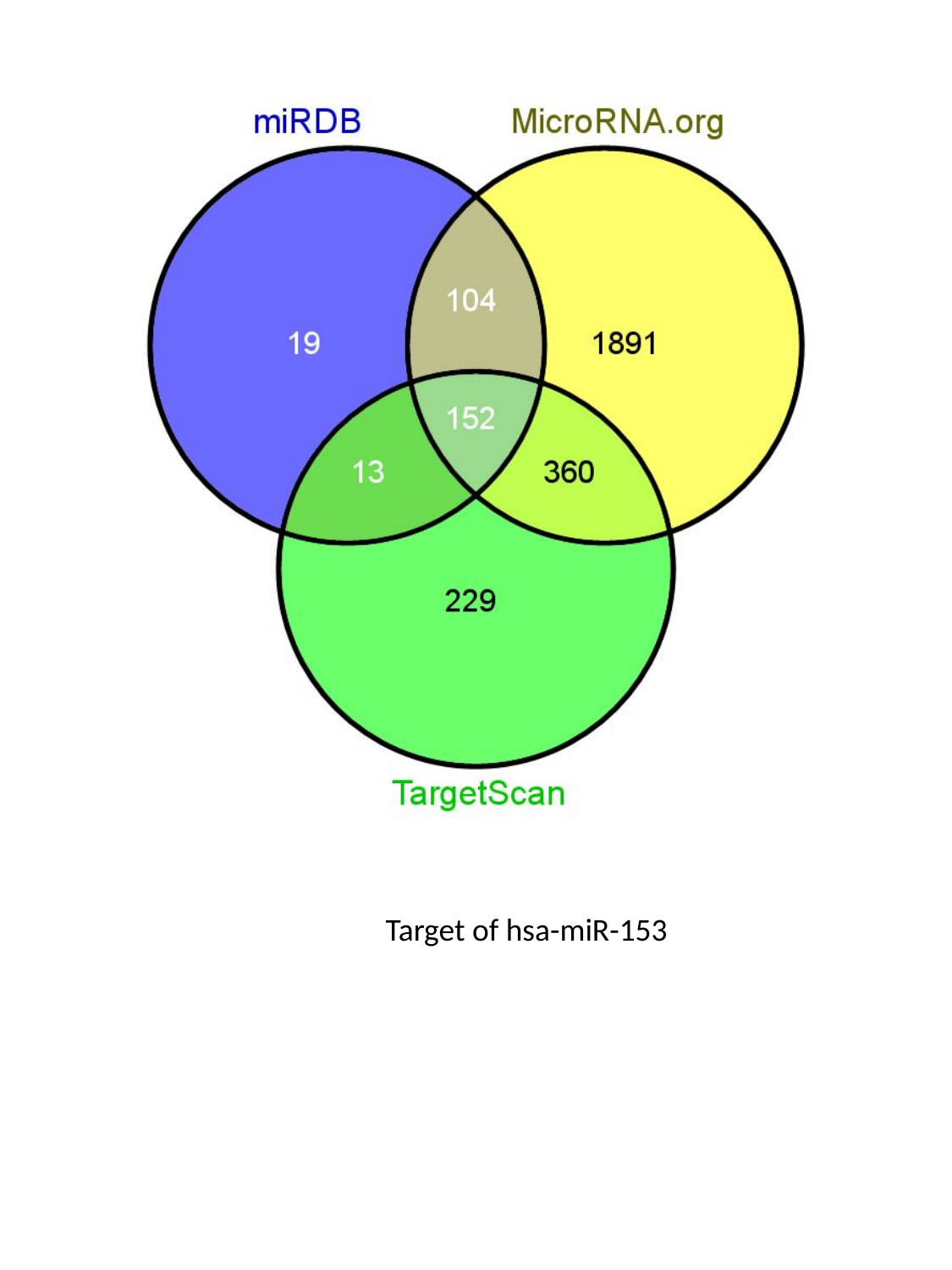

Target of hsa-miR-153

## Slide 3
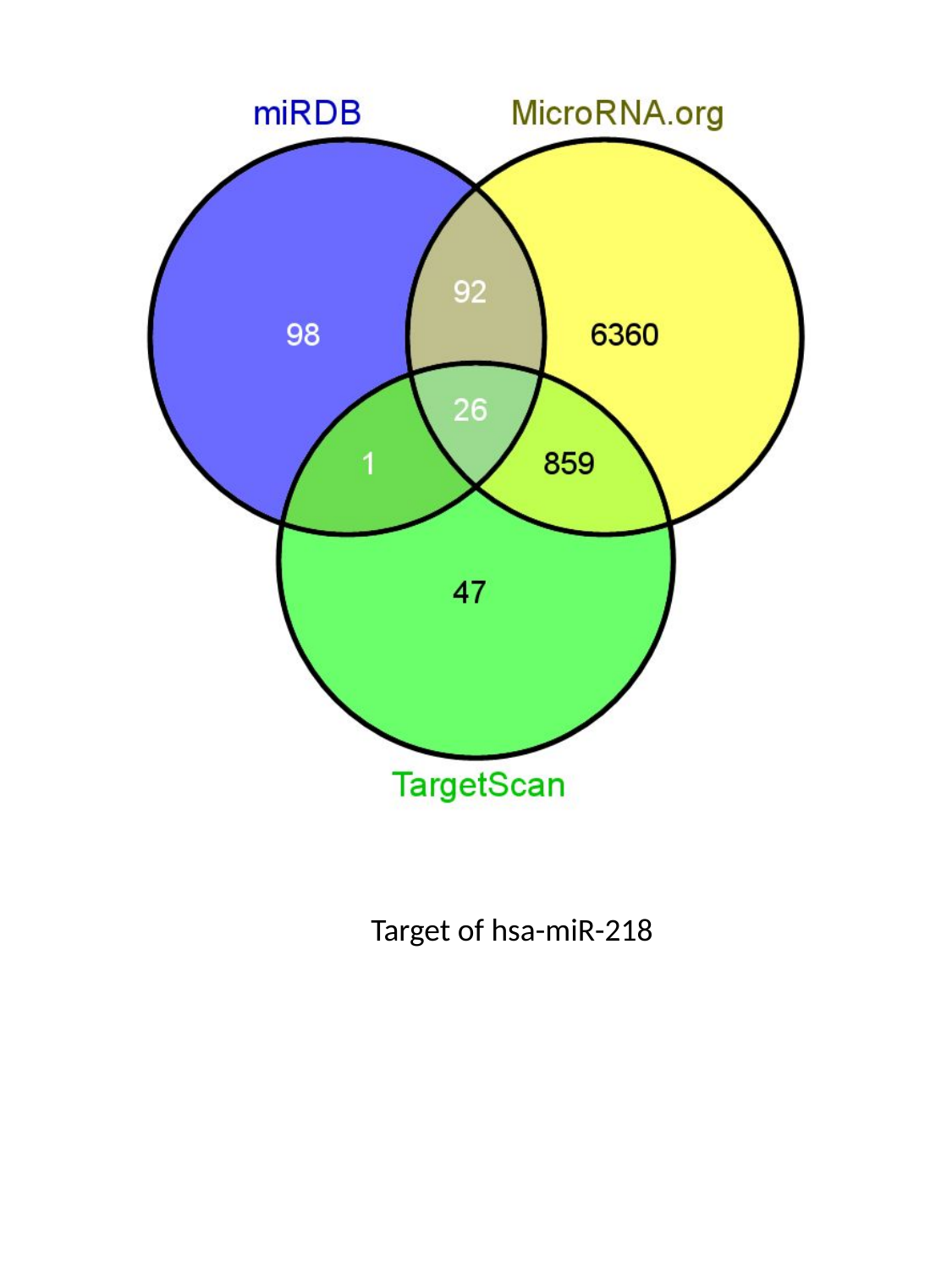

Target of hsa-miR-218

Supplement: Additional file 4 — Figure S2. Target of hsa-miR-137. [file 1471-2164-13-677-S4.pptx]

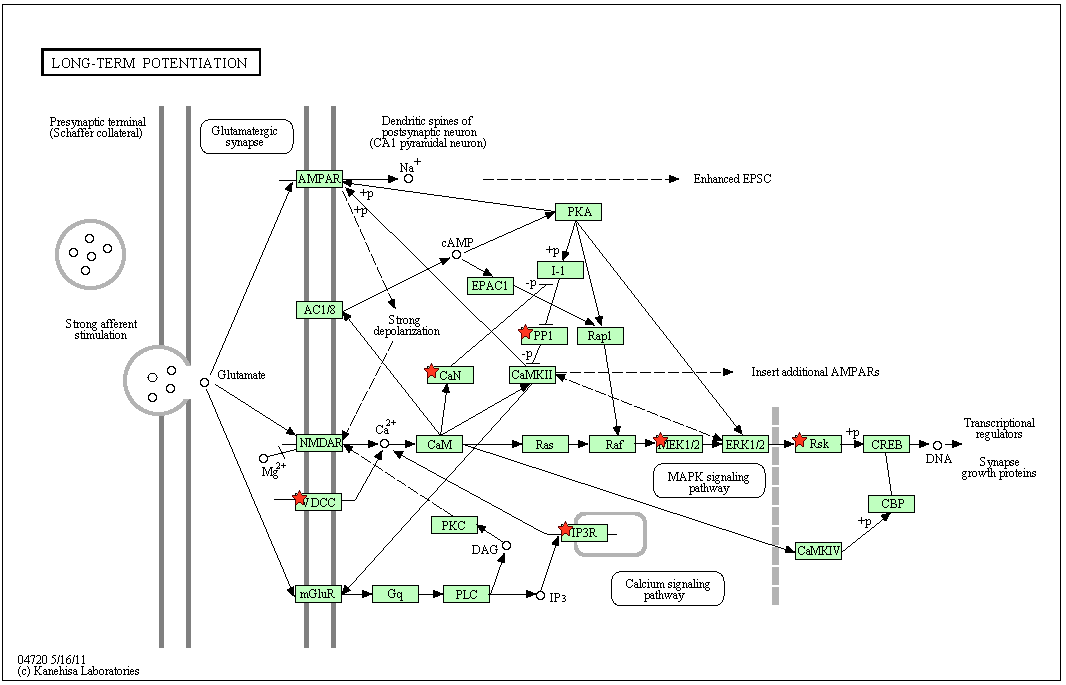

Supplement: Additional file 8 — Figure S3. Significantly dysregulated genes on long term potentiation pathway in HAD brains compared to HIV non-dementia brains. [file 1471-2164-13-677-S8.png]

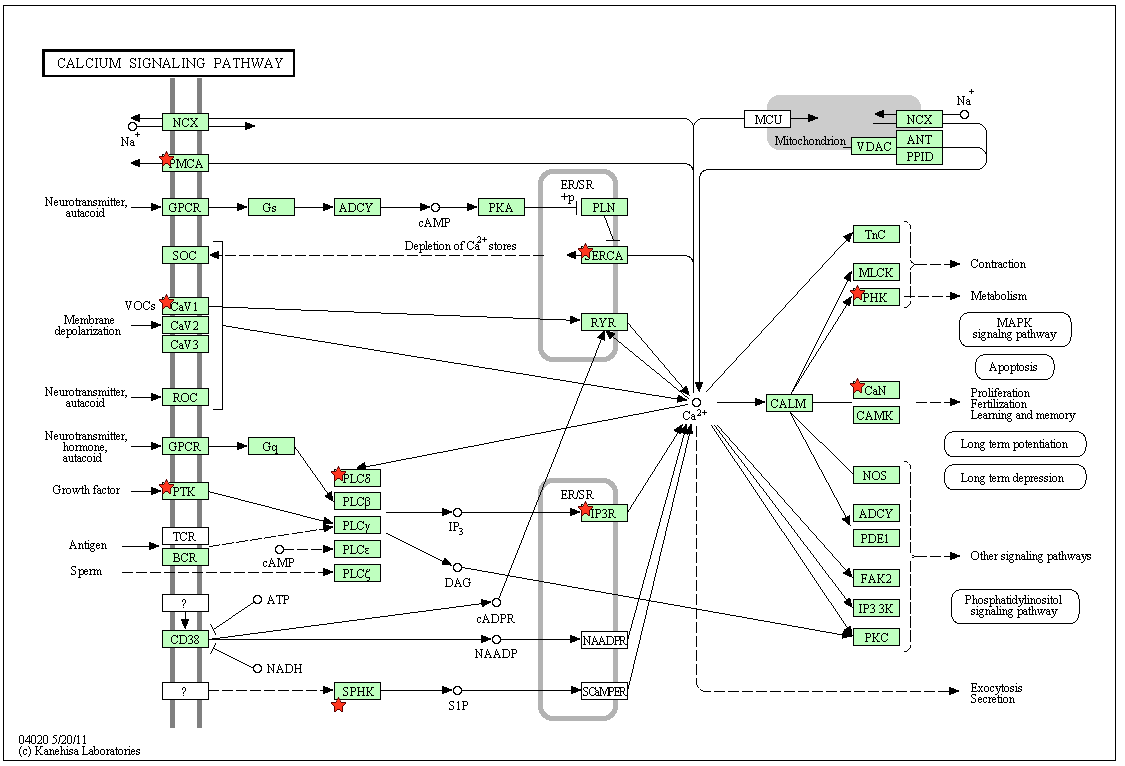

Supplement: Additional file 10 — Figure S5. Significantly dysregulated genes on calcium signalling pathway in HAD brains compared to HIV non-dementia brains. [file 1471-2164-13-677-S10.png]

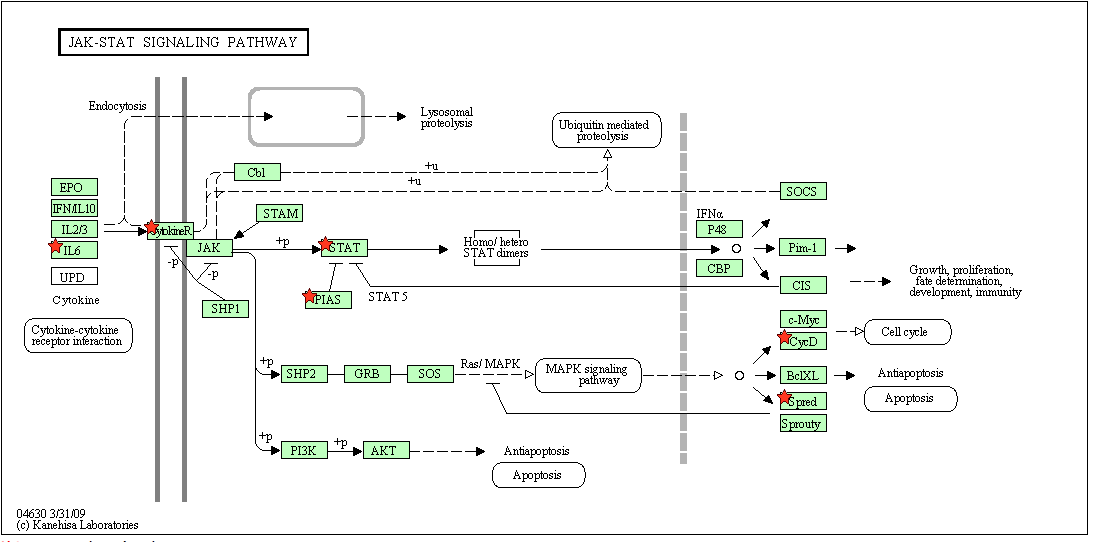

Supplement: Additional file 11 — Figure S6. Significantly dysregulated genes on Jak-STAT signalling pathway in HAD brains compared to HIV non-dementia brains. [file 1471-2164-13-677-S11.png]

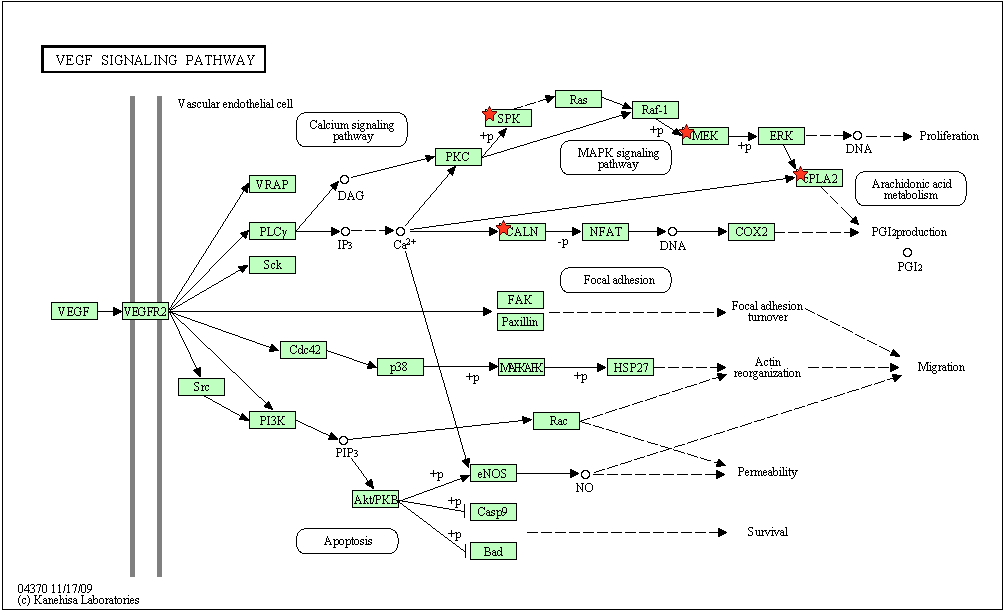

Supplement: Additional file 12 — Figure S7. Significantly dysregulated genes on VEGF signalling pathway in HAD brains compared to HIV non-dementia brains. [file 1471-2164-13-677-S12.png]
