# Supplementary material for: A parallel genome-wide mRNA and microRNA profiling of the frontal cortex of HIV patients with and without HIV-associated dementia shows the role of axon guidance and downstream pathways in HIV-mediated neurodegeneration
Source: BMC Genomics. 2012 Nov 28;13:677. doi: 10.1186/1471-2164-13-677 (PMC3560210; doi:10.1186/1471-2164-13-677)
Supplement: Additional file 6 — Table S4: Gene sets significantly enriched by GSEA analysis. [file 1471-2164-13-677-S6.docx]

**Additional file 6. Gene sets significantly enriched by GSEA analysis**

| NAME | Brief description |
| --- | --- |
| CYTOSKELETON_DEPENDENT_  INTRACELLULAR_TRANSPORT | Genes annotated by GO term GO:0030705. The directed movement of substances along cytoskeletal elements such as microfilaments or microtubules within a cell. |
| REGULATION_OF_BLOOD_PRESSURE | Genes annotated by the GO term GO:0008217. The process that modulates the force with which blood travels through the circulatory system. The process is controlled by a balance of processes that increase pressure and decrease pressure |
| ATPASE_ACTIVITY__COUPLED_TO_  TRANSMEMBRANE_MOVEMENT_OF_IONS__PHOSPHORYLATIVE_MECHANISM | Genes annotated by the GO term GO:0015662. Catalysis of the transfer of a solute or solutes from one side of a membrane to the other according to the reaction: ATP + H2O = ADP + phosphate, to directly drive the transport of ions across a membrane. The reaction is characterized by the transient formation of a high-energy aspartyl-phosphoryl-enzyme intermediate. |
| PROTEIN_HOMOOLIGOMERIZATION | Genes annotated by the GO term GO:0051260. The process of creating protein oligomers, compounds composed of a small number, usually between three and ten, of identical component monomers. Oligomers may be formed by the polymerization of a number of monomers or the depolymerization of a large protein polymer. |
| DENDRITE | Genes annotated by the GO term GO:0030425. A branching protoplasmic process of a neuron that receive and integrate signals coming from axons of other neurons, and convey the resulting signal to the body of the cell. |
| MRNA_BINDING | Genes annotated by the GO term GO:0003729. Interacting selectively with pre-messenger RNA (pre-mRNA) or messenger RNA (mRNA). |
| ATPASE_ACTIVITY__COUPLED_TO_  TRANSMEMBRANE_MOVEMENT_OF_IONS | Genes annotated by the GO term GO:0042625. Catalysis of the transfer of an ion from one side of a membrane to the other, driven by the reaction: ATP + H2O = ADP + phosphate. |
| FEEDING_BEHAVIOR | Genes annotated by the GO term GO:0007631. Behavior associated with the intake of food. |
