# Supplementary material for: A parallel genome-wide mRNA and microRNA profiling of the frontal cortex of HIV patients with and without HIV-associated dementia shows the role of axon guidance and downstream pathways in HIV-mediated neurodegeneration
Source: BMC Genomics. 2012 Nov 28;13:677. doi: 10.1186/1471-2164-13-677 (PMC3560210; doi:10.1186/1471-2164-13-677)
Supplement: Additional file 9 — Figure S4. Western blot validation of MAP2K2 and MAP2K4. MAP2K2 and MAP2K4 encoding proteins MEK2 (A) and JNK (B) with relative molecular weight 45 and 46kD respectively were separated on 12% SDSpolyacrylamide gel and blotting with specific antibodies against them. Semiquantitative analysis has been carried out by comparing the relative protein level (standardized by Actin (C)) in HAD and HIV non-dementia patients. The quantification result demonstrated the trend similar to the one observed in microarray and qPCR for HAD patients when compared to HIV non-dementia patients. [file 1471-2164-13-677-S9.pdf]

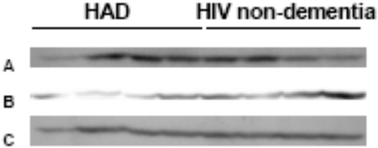

Additional file 9. Western blot validation of MAP2K2 and MAP2K4
MAP2K2 and MAP2K4 encoding proteins MEK2 (A) and JNK (B) with relative molecular weight 45 and 46kD respectively were separated on 12% SDS-polyacrylamide gel and blotting with specific antibodies against them. Semi-quantitative analysis has been carried out by comparing the relative protein level (standardized by Actin (C)) in HAD and HIV non-dementia patients. The quantification result demonstrated the trend similar to the one observed in microarray and qPCR for HAD patients when compared to HIV non-dementia patients.
