# Supplementary material for: A parallel genome-wide mRNA and microRNA profiling of the frontal cortex of HIV patients with and without HIV-associated dementia shows the role of axon guidance and downstream pathways in HIV-mediated neurodegeneration
Source: BMC Genomics. 2012 Nov 28;13:677. doi: 10.1186/1471-2164-13-677 (PMC3560210; doi:10.1186/1471-2164-13-677)
Supplement: Additional file 13 — Table S6. DE miRNAs and their target pathways. [file 1471-2164-13-677-S13.docx]

**Additional file13. DE miRNAs and their target pathways**

| **Pathways** | **miRNA targeting specific pathways** |
| --- | --- |
| **Neurodegeneration related pathways** | |
| Axon guidance | hsa-miR-219, hsa-miR-137, hsa-miR-218, hsa-miR-153, hsa-miR-323, hsa-miR-940, hsa-miR-338, hsa-miR-570, hsa-miR-19a |
| Long-term potentiation | hsa-miR-323, hsa-miR-495, hsa-miR-338, hsa-miR-216, hsa-miR-19a, hsa-miR-137, hsa-miR-218 |
| Neurotrophin signaling pathway | hsa-miR-940, hsa-miR-570, hsa-miR-19a, hsa-miR-137 |
| Long-term depression | hsa-miR-218, hsa-miR-323 |
| Amyotrophic lateral sclerosis (ALS) | hsa-miR-19a |
| **Signaling pathways** | |
| Wnt signaling pathway | hsa-miR-137, hsa-miR-153, hsa-miR-299, hsa-miR-323, hsa-miR-216, hsa-miR-570, hsa-miR-19a, hsa-miR-584, hsa-miR-219, hsa-miR-592 |
| Insulin signaling pathway | hsa-miR-19a, hsa-miR-219, hsa-miR-122, hsa-miR-137, hsa-miR-218, hsa-miR-153, hsa-miR-495 |
| mTOR signaling pathway | hsa-miR-137, hsa-miR-323, hsa-miR-495, hsa-miR-19a |
| TGF-beta signaling pathway | hsa-miR-570, hsa-miR-19a, hsa-miR-592, hsa-miR-153 |
| ErbB signaling pathway | hsa-miR-137, hsa-miR-218, hsa-miR-940, hsa-miR-483 |
| MAPK signaling pathway | hsa-miR-137, hsa-miR-940, hsa-miR-19a |
| Calcium signaling pathway | hsa-miR-19a, hsa-miR-137, hsa-miR-218 |
| GnRH signaling pathway | hsa-miR-137, hsa-miR-323, hsa-miR-19a |
| VEGF signaling pathway | hsa-miR-137, hsa-miR-940, |
| Adipocytokine signaling pathway | hsa-miR-137, hsa-miR-19a |
| Notch signaling pathway | hsa-miR-137, hsa-miR-495 |
| Fc epsilon RI signaling pathway | hsa-miR-19a |
| Hedgehog signaling pathway | hsa-miR-19a |
| Jak-STAT signaling pathway | hsa-miR-19a |
| p53 signaling pathway | hsa-miR-19a |
| Phosphatidylinositol signaling system | hsa-miR-137 |
| **Inflammation related pathways** |  |
| Endocytosis | hsa-miR-19a, hsa-miR-218, hsa-miR-153, hsa-miR-323, hsa-miR-495, hsa-miR-940, hsa-miR-483, hsa-miR-216, hsa-miR-570 |
| Fc gamma R-mediated phagocytosis | hsa-miR-218, hsa-miR-940 |
| Chemokine signaling pathway | hsa-miR-218, hsa-miR-19a |
| Leukocyte transendothelial migration | hsa-miR-218 |
| T cell receptor signaling pathway | hsa-miR-940 |
| B cell receptor signaling pathway | hsa-miR-940 |
| **Adhesion/junction pathways** | |
| Focal adhesion | hsa-miR-940, hsa-miR-483, hsa-miR-19a, hsa-miR-137, hsa-miR-218, hsa-miR-153, hsa-miR-495 |
| Adherens junction | hsa-miR-218, hsa-miR-153, hsa-miR-323, hsa-miR-495, hsa-miR-940 |
| Gap junction | hsa-miR-19a, hsa-miR-137, hsa-miR-218, hsa-miR-153, hsa-miR-323 |
| Tight junction | hsa-miR-218, hsa-miR-216 |
| Cell adhesion molecules (CAMs) | hsa-miR-495 |
| **Cellular processes pathways** | |
| Apoptosis | hsa-miR-940 |
| Cell cycle | hsa-miR-570 |
| Regulation of actin cytoskeleton | hsa-miR-153, hsa-miR-338, hsa-miR-19a, hsa-miR-376 |
| **Genetic information processing pathways** | |
| Ubiquitin mediated proteolysis | hsa-miR-218, hsa-miR-153, hsa-miR-323, hsa-miR-495, hsa-miR-219 |
| Spliceosome | hsa-miR-570 |
| SNARE interactions in vesicular transport | hsa-miR-19a |
| **Metabolism pathways** |  |
| Heparan sulfate biosynthesis | hsa-miR-218, hsa-miR-153 |
| Glycolysis / Gluconeogenesis | hsa-miR-122 |
| **Cancer related pathways** | |
| Pathways in cancer | hsa-miR-218, hsa-miR-153, hsa-miR-323, hsa-miR-495, hsa-miR-940, hsa-miR-483, hsa-miR-216, hsa-miR-570, hsa-miR-219, hsa-miR-376 |
| Glioma | hsa-miR-495, hsa-miR-483, hsa-miR-570, hsa-miR-19a, hsa-miR-137, hsa-miR-218, hsa-miR-153 |
| Colorectal cancer | hsa-miR-153, hsa-miR-323, hsa-miR-940, hsa-miR-216, hsa-miR-19a, hsa-miR-219 |
| Melanoma | hsa-miR-570, hsa-miR-19a, hsa-miR-376, hsa-miR-153, hsa-miR-495 |
| Prostate cancer | hsa-miR-153, hsa-miR-495, hsa-miR-570 |
| Chronic myeloid leukemia | hsa-miR-218, hsa-miR-495 |
| Endometrial cancer | hsa-miR-153, hsa-miR-19a |
| Pancreatic cancer | hsa-miR-137, hsa-miR-495 |
| Renal cell carcinoma | hsa-miR-940, hsa-miR-19a |
| Acute myeloid leukemia | hsa-miR-940 |
| Basal cell carcinoma | hsa-miR-219 |
| Non-small cell lung cancer | hsa-miR-495 |
| Small cell lung cancer | hsa-miR-495 |
| **Others** | |
| Hypertrophic cardiomyopathy (HCM) | hsa-miR-137, hsa-miR-19a |
| Arrhythmogenic right ventricular cardiomyopathy (ARVC) | hsa-miR-19a, hsa-miR-137 |
| Dilated cardiomyopathy | hsa-miR-19a, hsa-miR-137 |
| Aldosterone-regulated sodium reabsorption | hsa-miR-19a, hsa-miR-495 |
| Dorso-ventral axis formation | hsa-miR-940, hsa-miR-570, hsa-miR-940 |
| ECM-receptor interaction | hsa-miR-19a |
| Melanogenesis | hsa-miR-137, hsa-miR-218, hsa-miR-323, hsa-miR-19a |
| Oocyte meiosis | hsa-miR-137, hsa-miR-218, hsa-miR-323, hsa-miR-940, hsa-miR-19a |
| Progesterone-mediated oocyte maturation | hsa-miR-19a, hsa-miR-218, hsa-miR-495 |
| Type II diabetes mellitus | hsa-miR-19a, hsa-miR-376, |
| Vascular smooth muscle contraction | hsa-miR-137, hsa-miR-218, hsa-miR-323, hsa-miR-19a |
