# Supplementary material for: A parallel genome-wide mRNA and microRNA profiling of the frontal cortex of HIV patients with and without HIV-associated dementia shows the role of axon guidance and downstream pathways in HIV-mediated neurodegeneration
Source: BMC Genomics. 2012 Nov 28;13:677. doi: 10.1186/1471-2164-13-677 (PMC3560210; doi:10.1186/1471-2164-13-677)
Supplement: Additional file 14 — Table S7. Patient details. [file 1471-2164-13-677-S14.docx]

| Sample ID | sex^a^ | Age at death | HAD stage^b^ | ART (ARV) | Non-HIVE neuropathology | Duration from seropositive for HIV to death(year) | Postmortem interval (h) | Study  Involved in |
| --- | --- | --- | --- | --- | --- | --- | --- | --- |
| 1 | M | 38 | L | N/A | None | 12 | N/A | M, Mi and W |
| 2 | M | 45 | L |  | N/A | N/A | N/A | M |
| 3 | M | 63 | L |  | N/A | N/A | N/A | M and W |
| 4 | M | 23 | L | AZT, DDI | N/A | 3/4 | N/A | M and Mi |
| 5 | M | 42 | E | N/A | Leukoencephalopathy | 19 | 39 | M and Mi |
| 6 | M | 62 | E |  | N/A | N/A | N/A | M |
| 7 | M | 32 | E |  | None | 9 | 14.5 | M and Mi |
| 8 | M | N/A | E |  | N/A | N/A | N/A | M and W |
| 9 | M | N/A | E |  | N/A | N/A | N/A | M and W |
| 10 | M | N/A | E |  | N/A | N/A | N/A | M and Mi |
| 11 | M | 49 | N | NVP, NFV, ZDV, SQV, D4T, 3TC, FTV, DDI, RTV, NVP, ABC, CBV, APV, TZV, KTA | Minimal non-diagnostic abnormalities | 10 | 16 | M |
| 12 | M | 67 | N |  | None | N/A | 15 | M, Mi and W |
| 13 | M | 45 | N | 3TC, ABC, KTA, NFV, RTV, TFV | None | 21 | 12 | M, Mi and W |
| 14 | M | 55 | N | 3TC, DLV, TFV, NFV | None | 11 | 120 | W |
| 15 | M | 35 | N |  | N/A | N/A | N/A | M |
| 16 | M | 53 | N | 3TC, APV, EFV, ZDV, RTV | None | 22 | <24 | M, Mi and W |
| 17 | M | 59 | N | 3TC, D4T, KTA, RTV, TFV | None | 13 | 12 | M and Mi |
| 18 | F | 52 | N | 3TC, D4T, DDI, KTA, RTV | None | 21 | 8 | M and Mi |

**Additional file14. Patient details**

^a^ M, male; F, female

^b^ HAD: HIV associated dementia; L: late stage; E: early stage; N: HIV non-dementia

N/A: no data available

AZT: zidovudine, ddl: didanosine, NVP: nevirapine, NFV: nelfinavir, ZDV: zidovudine, SQV: saquinavir, D4T: stavudine, 3TC: 2'-deoxy-3'-thiacytidine, FTV: fortovase, RTV:ritonavir,

ABC: abacavir, CBV: combivir, APV: amprenavir, TZV: trizivir, TFV: tenofovir, DLV: delavirdine, Blank cells: No treatment.

M: microarray and qPCR validation

Mi: miRNA array and miRNA qPCR validation

W: western blot
