# Supplementary material for: A parallel genome-wide mRNA and microRNA profiling of the frontal cortex of HIV patients with and without HIV-associated dementia shows the role of axon guidance and downstream pathways in HIV-mediated neurodegeneration
Source: BMC Genomics. 2012 Nov 28;13:677. doi: 10.1186/1471-2164-13-677 (PMC3560210; doi:10.1186/1471-2164-13-677)
Supplement: Additional file 15 — Table S8. qPCR primers. [file 1471-2164-13-677-S15.doc]

**Additional file15. qPCR primers**

| Gene symbol | Primers | |
| --- | --- | --- |
| AQP1 | left primer | gtccaggacaacgtgaaggt |
|  | right primer | gaggaggtgatgcctgagag |
| HBB | left primer | gcaacctcaaacagacacca |
|  | right primer | cagcatcaggagtggacaga |
| MOBP | left primer | gcaacctcaaacagacacca |
|  | right primer | cagcatcaggagtggacaga |
| SPATA13 | left primer | ttcctgaagactcggttgct |
|  | right primer | catacaagctgaccccacct |
| CALD1 | left primer | tgcagaaaagcagtggtgtc |
|  | right primer | ccttcagcaggaacaggaag |
| ATP8A2 | left primer | gtcctgggagaaagggaaag |
|  | right primer | aggagagcaacctgcctaca |
| ATXN3 | left primer | agctgagcacacactggatg |
|  | right primer | cttgcactggcatcttttca |
| DSEL (C18orf4) | left primer | gcccagtcattagcccataa |
|  | right primer | caggcaccacctccactatt |
| ADAM22 | left primer | ttacccaatgcctccacttc |
|  | right primer | tgtgaaactccgcttcctct |
| CCL8 | left primer | tcacctgctgctttaacgtg |
|  | right primer | atccctgacccatctctcct |
| UBE2D2 | left primer | gatcacagtggtctccagca |
|  | right primer | tccattcccgagctattctg |
